# Stochastic Computations in Cortical Microcircuit Models

**DOI:** 10.1371/journal.pcbi.1003311

**Published:** 2013-11-14

**Authors:** Stefan Habenschuss, Zeno Jonke, Wolfgang Maass

**Affiliations:** Graz University of Technology, Institute for Theoretical Computer Science, Graz, Austria; Indiana University, United States of America

## Abstract

Experimental data from neuroscience suggest that a substantial amount of knowledge is stored in the brain in the form of probability distributions over network states and trajectories of network states. We provide a theoretical foundation for this hypothesis by showing that even very detailed models for cortical microcircuits, with data-based diverse nonlinear neurons and synapses, have a stationary distribution of network states and trajectories of network states to which they converge exponentially fast from any initial state. We demonstrate that this convergence holds in spite of the non-reversibility of the stochastic dynamics of cortical microcircuits. We further show that, in the presence of background network oscillations, separate stationary distributions emerge for different phases of the oscillation, in accordance with experimentally reported phase-specific codes. We complement these theoretical results by computer simulations that investigate resulting computation times for typical probabilistic inference tasks on these internally stored distributions, such as marginalization or marginal maximum-a-posteriori estimation. Furthermore, we show that the inherent stochastic dynamics of generic cortical microcircuits enables them to quickly generate approximate solutions to difficult constraint satisfaction problems, where stored knowledge and current inputs jointly constrain possible solutions. This provides a powerful new computing paradigm for networks of spiking neurons, that also throws new light on how networks of neurons in the brain could carry out complex computational tasks such as prediction, imagination, memory recall and problem solving.

## Author Summary

The brain has not only the capability to process sensory input, but it can also produce predictions, imaginations, and solve problems that combine learned knowledge with information about a new scenario. But although these more complex information processing capabilities lie at the heart of human intelligence, we still do not know how they are organized and implemented in the brain. Numerous studies in cognitive science and neuroscience conclude that many of these processes involve probabilistic inference. This suggests that neuronal circuits in the brain process information in the form of probability distributions, but we are missing insight into how complex distributions could be represented and stored in large and diverse networks of neurons in the brain. We prove in this article that realistic cortical microcircuit models can store complex probabilistic knowledge by embodying probability distributions in their inherent stochastic dynamics – yielding a knowledge representation in which typical probabilistic inference problems such as marginalization become straightforward readout tasks. We show that in cortical microcircuit models such computations can be performed satisfactorily within a few . Furthermore, we demonstrate how internally stored distributions can be programmed in a simple manner to endow a neural circuit with powerful problem solving capabilities.

## Introduction

The question whether brain computations are inherently deterministic or inherently stochastic is obviously of fundamental importance. Numerous experimental data highlight inherently stochastic aspects of neurons, synapses and networks of neurons on virtually all spatial and temporal scales that have been examined [1]–[5]. A clearly visible stochastic feature of brain activity is the trial-to-trial variability of neuronal responses, which also appears on virtually every spatial and temporal scale that has been examined [2]. This variability has often been interpreted as side-effect of an implementation of inherently deterministic computing paradigms with noisy elements, and it has been attempted to show that the observed noise can be eliminated through spatial or temporal averaging. However, more recent experimental methods, which make it possible to record simultaneously from many neurons (or from many voxels in fMRI), have shown that the underlying probability distributions of network states during spontaneous activity are highly structured and multimodal, with distinct modes that resemble those encountered during active processing. This has been shown through recordings with voltage-sensitive dyes starting with [6], [7], multi-electrode arrays [8], and fMRI [9], [10]. It was also shown that the intrinsic trial-to-trial variability of brain systems is intimately related to the observed trial-to-trial variability in behavior (see e.g. [11]). Furthermore, in [12] it was shown that during navigation in a complex environment where simultaneously two spatial frames of reference were relevant, the firing of neurons in area *CA1* represented both frames in alternation, so that coactive neurons tended to relate to a common frame of reference. In addition it has been shown that in a situation where sensory stimuli are ambiguous, large brain networks switch stochastically between alternative interpretations or percepts, see [13]–[15]. Furthermore, an increase in the volatility of network states has been shown to accompany episodes of behavioral uncertainty [16]. All these experimental data point to inherently stochastic aspects in the organization of brain computations, and more specifically to an important computational role of spontaneously varying network states of smaller and larger networks of neurons in the brain. However, one should realize that the approach to stochastic computation that we examine in this article does not postulate that all brain activity is stochastic or unreliable, since reliable neural responses can be represented by probabilities close to 1.

The goal of this article is to provide a theoretical foundation for understanding stochastic computations in networks of neurons in the brain, in particular also for the generation of structured spontaneous activity. To this end, we prove here that even biologically realistic models 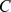 for networks of neurons in the brain have – for a suitable definition of network state – a unique stationary distribution 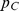 of network states. Previous work had focused in this context on neuronal models with linear sub-threshold dynamics [17], [18] and constant external input (e.g. constant input firing rates). However, we show here that this holds even for quite realistic models that reflect, for example, data on nonlinear dendritic integration (dendritic spikes), synapses with data-based short term dynamics (i.e., individual mixtures of depression and facilitation), and different types of neurons on specific laminae. We also show that these results are not restricted to the case of constant external input, but rather can be extended to periodically changing input, and to input generated by arbitrary ergodic stochastic processes.

Our theoretical results imply that virtually any data-based model 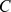, for networks of neurons featuring realistic neuronal noise sources (e.g. stochastic synaptic vesicle release) implements a Markov process through its stochastic dynamics. This can be interpreted – in spite of its non-reversibility – as a form of sampling from a unique stationary distribution 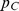. One interpretation of 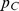, which is in principle consistent with our findings, is that it represents the posterior distribution of a Bayesian inference operation [19]–[22], in which the current input (evidence) is combined with prior knowledge encoded in network parameters such as synaptic weights or intrinsic excitabilities of neurons (see [23]–[26] for an introduction to the “Bayesian brain”). This interpretation of neural dynamics as sampling from a posterior distribution is intriguing, as it implies that various results of probabilistic inference could then be easily obtained by a simple readout mechanism: For example, posterior marginal probabilities can be estimated (approximately) by observing the number of spikes of specific neurons within some time window (see related data from parietal cortex [27]). Furthermore, an approximate maximal a posteriori (MAP) inference can be carried out by observing which network states occur more often, and/or are more persistent.

A crucial issue which arises is whether reliable readouts from 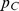 in realistic cortical microcircuit models can be obtained quickly enough to support, e.g., fast decision making in downstream areas. This critically depends on the speed of convergence of the distribution of network states (or distribution of trajectories of network states) from typical initial network states to the stationary distribution. Since the initial network state of a cortical microcircuit 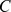 depends on past activity, it may often be already quite “close” to the stationary distribution when a new input arrives (since past inputs are likely related to the new input). But it is also reasonable to assume that the initial state of the network is frequently unrelated to the stationary distribution 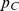, for example after drastic input changes. In this case the time required for readouts depends on the expected convergence speed to 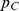 from – more or less – *arbitrary* initial states. We show that one can prove exponential upper bounds for this convergence speed. But even that does not guarantee fast convergence for a concrete system, because of constant factors in the theoretical upper bound. Therefore we complement this theoretical analysis of the convergence speed by extensive computer simulations for cortical microcircuit models.

The notion of a cortical microcircuit arose from the observation that “it seems likely that there is a basically uniform microcircuit pattern throughout the neocortex upon which certain specializations unique to this or that cortical area are superimposed” [28]. This notion is not precisely defined, but rather a term of convenience: It refers to network models that are sufficiently large to contain examples of the main types of experimentally observed neurons on specific laminae, and the main types of experimentally observed synaptic connections between different types of neurons on different laminae, ideally in statistically representative numbers [29]. Computer simulations of cortical microcircuit models are practically constrained both by a lack of sufficiently many consistent data from a single preparation and a single cortical area, and by the available computer time. In the computer simulations for this article we have focused on a relatively simple standard model for a cortical microcircuit in the somatosensory cortex [30] that has already been examined in some variations in previous studies from various perspectives [31]–[34].

We show that for this standard model of a cortical microcircuit marginal probabilities for single random variables (neurons) can be estimated through sampling even for fairly large instances with 5000 neurons within a few 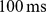 of simulated biological time, hence well within the range of experimentally observed computation times of biological organisms. The same holds for probabilities of network states for small sub-networks. Furthermore, we show that at least for sizes up to 5000 neurons these “computation times” are virtually independent of the size of the microcircuit model.

We also address the question to which extent our theoretical framework can be applied in the context of periodic input, for example in the presence of background theta oscillations [35]. In contrast to the stationary input case, we show that the presence of periodic input leads to the emergence of unique *phase-specific* stationary distributions, i.e., a separate unique stationary distribution for each phase of the periodic input. We discuss basic implications of this result and relate our findings to experimental data on theta-paced path sequences [35], [36] and bi-stable activity [37] in hippocampus.

Finally, our theoretically founded framework for stochastic computations in networks of spiking neurons also throws new light on the question how complex constraint satisfaction problems could be solved by cortical microcircuits [38], [39]. We demonstrate this in a toy example for the popular puzzle game Sudoku. We show that the constraints of this problem can be easily encoded by synaptic connections between excitatory and inhibitory neurons in such a way that the stationary distribution 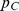 assigns particularly high probability to those network states which encode correct (or good approximate) solutions to the problem. The resulting network dynamics can also be understood as parallel stochastic search with anytime computing properties: Early network states provide very fast heuristic solutions, while later network states are distributed according to the stationary distribution 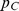, therefore visiting with highest probability those solutions which violate only a few or zero constraints.

In order to make the results of this article accessible to non-theoreticians we present in the subsequent Results section our main findings in a less technical formulation that emphasizes relationships to experimental data. Rigorous mathematical definitions and proofs can be found in the Methods section, which has been structured in the same way as the Results section in order to facilitate simultaneous access on different levels of detail.

## Results

### Network states and distributions of network states

**Figure 1:**
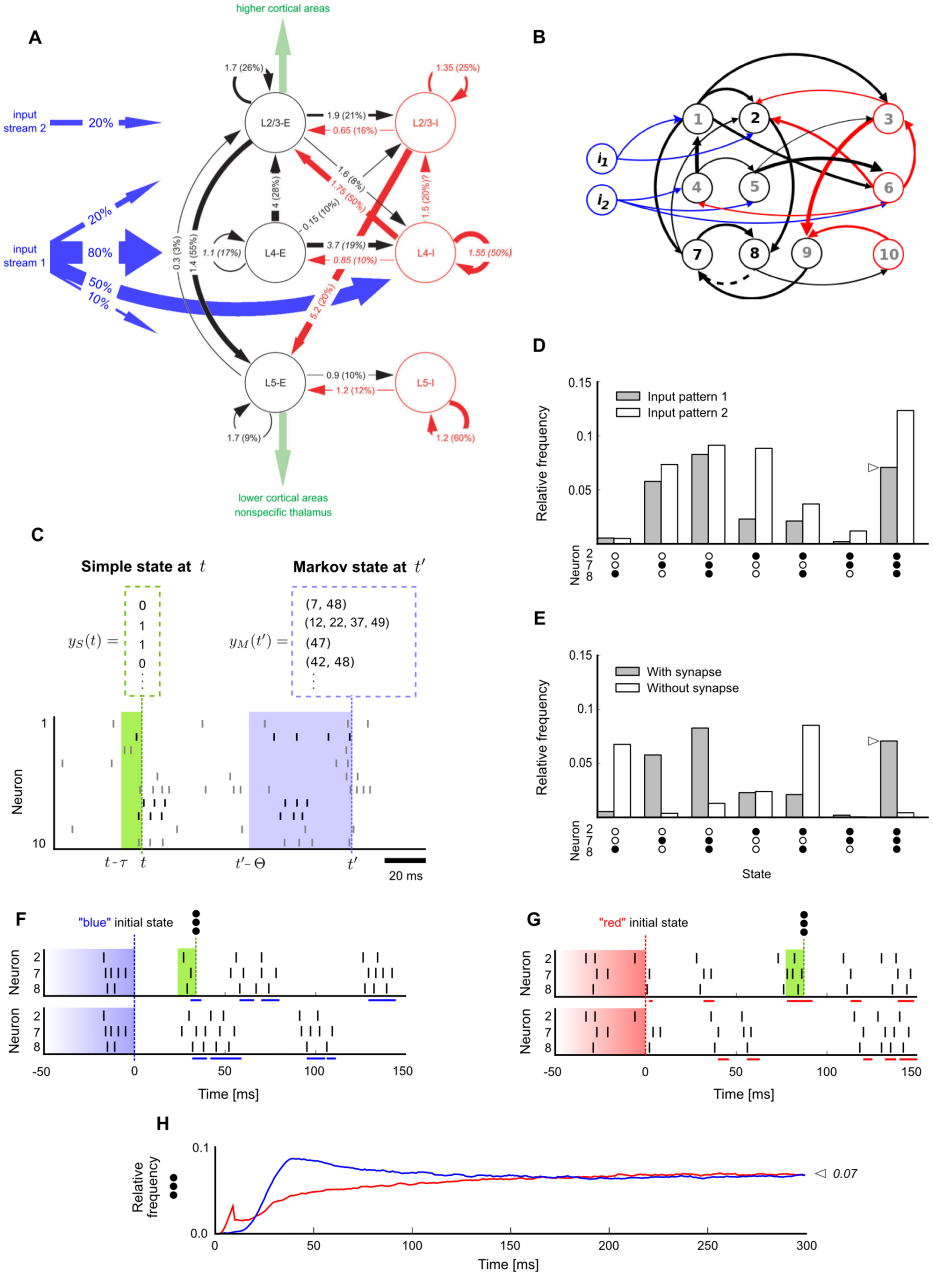
Network states and stationary distributions of network states in a cortical microcircuit model. **A**. Data-based cortical microcircuit template from Cereb. Cortex (2007) 17: 149-162 [30];  reprinted by permission of the authors and Oxford University Press. **B**. A small instantiation of this model consisting of 10 network neurons 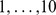 and 2 additional input neurons 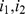. Neurons are colored by type (blue:input, black:excitatory, red:inhibitory). Line width represents synaptic efficacy. The synapse from neuron 8 to 7 is removed for the simulation described in E. **C**. Notions of network state considered in this article. Markov states are defined by the exact timing of all recent spikes within some time window 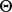, shown here for 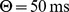. Simple states only record which neurons fired recently (0 = no spike, 1 = at least one spike within a short window 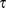, with 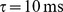 throughout this figure). **D**. Empirically measured stationary distribution of simple network states. Shown is the marginal distribution 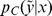 for a subset of three neurons 2,7,8 (their spikes are shown in C in black), under two different input conditions (input pattern 1: 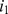 firing at 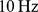 and 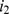 at 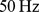, input pattern 2: 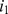 at 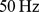 and 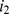 at 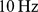). The distribution for each input condition was obtained by measuring the relative time spent in each of the simple states (0,0,0), …, (1,1,1) in a single long trial (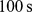). The zero state (0,0,0) is not shown. **E**. Effect of removing one synapse, from neuron 8 to neuron 7, on the stationary distribution of network states (input pattern 1 was presented). **F**. Illustration of trial-to-trial variability in the small cortical microcircuit (input pattern 1). Two trials starting from identical initial network states 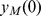 are shown. Blue bars at the bottom of each trial mark periods where the subnetwork of neurons 2,7,8 was in simple state (1,1,1) at this time 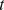. Note that the “blue” initial Markov state is shown only partially: it is actually longer and comprises all neurons in the network (as in panel C, but with 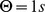). **G**. Two trials starting from a different (“red”) initial network state. Red bars denote periods of state (1,1,1) for “red” trials. **H**. Convergence to the stationary distribution 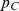 in this small cortical microcircuit is fast and independent of the initial state: This is illustrated for the relative frequency of simple state (1,1,1) within the first 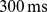 after input onset. The blue/red line shows the relative frequency of simple state (1,1,1) at each time 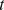 estimated from many (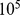) “blue”/“red” trials. The relative frequency of simple state (1,1,1) rapidly converges to its stationary value denoted by the symbol 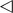 (marked also in panels D and E). The relative frequency converges to the same value regardless of the initial state (blue/red).

A simple notion of network state at time 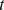 simply indicates which neurons in the network fired within some short time window before 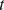. For example, in [20] a window size of 2ms was selected. However, the full network state could not be analyzed there experimentally, only its projection onto 16 electrodes in area V1 from which recordings were made. An important methodological innovation of [20] was to analyze under various conditions the probability distribution of the recorded fragments of network states, i.e., of the resulting bit vectors of length 16 (with a “1” at position 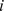 if a spike was recorded during the preceding 2ms at electrode 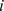). In particular, it was shown that during development the distribution over these 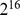 network states during spontaneous activity in darkness approximates the distribution recorded during natural vision. Apart from its functional interpretation, this result also raises the even more fundamental question how a network of neurons in the brain can represent and generate a complex distribution of network states. This question is addressed here in the context of data-based models 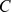 for cortical microcircuits. We consider notions of network states 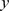 similar to [20] (see the simple state 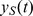 in Figure 1C) and provide a rigorous proof that under some mild assumptions any such model 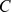 represents and generates for different external inputs 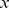 associated different internal distributions 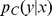 of network states 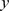. More precisely, we will show that for any specific input 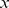 there exists a unique stationary distribution 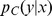 of network states 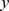 to which the network converges exponentially fast from any initial state.

This result can be derived within the theory of Markov processes on general state spaces, an extension of the more familiar theory of Markov chains on finite state spaces to continuous time and infinitely many network states. Another important difference to typical Markov chains (e.g. the dynamics of Gibbs sampling in Boltzmann machines) is that the Markov processes describing the stochastic dynamics of cortical microcircuit models are non-reversible. This is a well-known difference between simple neural network models and networks of spiking neurons in the brain, where a spike of a neuron causes postsynaptic potentials in other neurons - but not vice versa. In addition, experimental results show that brain networks tend to have a non-reversible dynamics also on longer time scales (e.g., stereotypical trajectories of network states [40]–[43]).

In order to prove results on the existence of stationary distributions 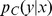 of network states 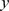, one first needs to consider a more complex notion of network state 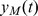 at time 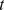, which records the history of all spikes in the network 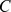 since time 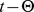 (see Figure 1C). The window length 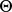 has to be chosen sufficiently large so that the influence of spikes before time 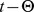 on the dynamics of the network after time 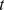 can be neglected. This more complex notion of network state then fulfills the *Markov property*, such that the future network evolution depends on the past only through the current Markov state. The existence of a window length 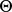 with the Markov property is a basic assumption of the subsequent theoretical results. For standard models of networks of spiking neurons a value of 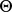 around 100ms provides already a good approximation of the Markov property, since this is a typical time during which a post-synaptic potential has a non-negligible effect at the soma of a post-synaptic neuron. For more complex models of networks of spiking neurons a larger value of 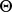 in the range of seconds is more adequate, in order to accommodate for dendritic spikes or the activation of 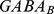 receptors that may last 100ms or longer, and the short term dynamics of synapses with time constants of several hundred milliseconds. Fortunately, once the existence of a stationary distribution is proved for such more complex notion of network state, it also holds for any simpler notion of network state (even if these simpler network states do not fulfill the Markov property), that results when one ignores details of the more complex network states. For example, one can ignore all spikes before time 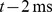, the exact firing times within the window from 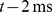 to 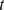, and whether a neuron fired one or several spikes. In this way one arrives back at the simple notion of network state from [20].

#### Theorem 1 (Exponentially fast convergence to a stationary distribution)

*Let* 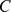 *be an arbitrary model for a network of spiking neurons with stochastic synaptic release or some other mechanism for stochastic firing.* 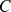 *may consist of complex multi-compartment neuron models with nonlinear dendritic integration (including dendritic spikes) and heterogeneous synapses with differential short term dynamics. We assume that this network* 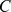 *receives external inputs from a set of input neurons* 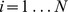 *which fire according to Poisson processes at different rates* 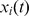 *. The vector* 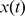 *of input rates can be either constant over time (* 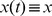 *), or generated by any external Markov process that converges exponentially fast to a stationary distribution.*

*Then there exists a stationary distribution* 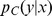 *of network states* 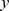 *, to which the stochastic dynamics of* 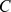 *converges from any initial state of the network exponentially fast. Accordingly, the distribution of subnetwork states* 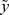 *of any subset of neurons converges exponentially fast to the marginal distribution* 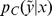 *of this subnetwork.*

Note that Theorem 1 states that the network embodies not only the joint distribution 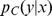 over all neurons, but simultaneously all marginal distributions 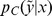 over all possible subsets of neurons. This property follows naturally from the fact that 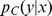 is represented in a sample-based manner [25]. As a consequence, if one is interested in estimating the marginal distribution of some subset of neurons rather than the full joint distribution, it suffices to observe the activity of the particular subnetwork of interest (while ignoring the remaining network). This is remarkable insofar, as the exact computation of marginal probabilities is in general known to be quite difficult (even NP-complete [44]).

Theorem 1 requires that neurons fire stochastically. More precisely, a basic assumption required for Theorem 1 is that the network behaves sufficiently stochastic at *any point in time*, in the sense that the probability that a neuron fires in an interval 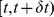 must be smaller than 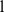 for any 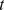. This is indeed fulfilled by any stochastic neuron model as long as instantaneous firing rates remain bounded. It is also fulfilled by any deterministic neuron model if synaptic transmission is modeled via stochastic vesicle release with bounded release rates. Another assumption is that long-term plasticity and other long-term memory effects have a negligible impact on the network dynamics on shorter timescales which are the focus of this article (milliseconds to a few seconds). Precise mathematical definitions of all assumptions and notions involved in Theorem 1 as well as proofs can be found in Methods (see Lemma 2 and 3).

An illustration for Theorem 1 is given in Figure 1. We use as our running example for a cortical microcircuit model 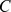 the model of [30] shown in Figure 1A, which consists of three populations of excitatory and three populations of inhibitory neurons on specific laminae. Average strength of synaptic connections (measured as mean amplitude of postsynaptic potentials at the soma in 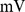, and indicated by the numbers at the arrows in Figure 1A) as well as the connection probability (indicated in parentheses at each arrow as 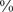 in Figure 1A) are based in this model on intracellular recordings from 998 pairs of identified neurons from the Thomson Lab [45]. The thickness of arrows in Figure 1A reflects the products of those two numbers for each connection. The nonlinear short-term dynamics of each type of synaptic connection was modeled according to data from the Markram Lab [46], [47]. Neuronal integration and spike generation was modeled by a conductance-based leaky-integrate-and-fire model, with a stochastic spiking mechanism based on [48]. See Methods for details.

The external input 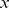 consists in a cortical microcircuit of inputs from higher cortical areas that primarily target neurons in superficial layers, and bottom-up inputs that arrive primarily in layer 4, but also on other layers (details tend to depend on the cortical area and the species). We model two input streams in a qualitative manner as in [30]. Also background synaptic input is modeled according to [30].

Figure 1B shows a small instantiation of this microcircuit template consisting of 10 neurons (we had to manually tune a few connections in this circuit to facilitate visual clarity of subsequent panels). The impact of different external inputs 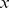 and of a single synaptic connection from neuron 8 to neuron 7 on the stationary distribution is shown in Figure 1D and E, respectively (shown is the marginal distribution 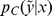 of a subset of three neurons 2,7 and 8). This illustrates that the structure and dynamics of a circuit 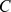 are intimately linked to properties of its stationary distribution 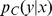. In fact, we argue that the stationary distribution 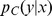 (more precisely: the stationary distribution 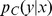 for all relevant external inputs 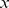) can be viewed as a mathematical model for the most salient aspects of stochastic computations in a circuit 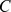.

The influence of the initial network state on the first 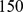 ms of network response is shown in Figure 1F and G for representative trials starting from two different initial Markov states (blue/red, two trials shown for each). Variability among trials arises from the inherent stochasticity of neurons and the presence of background synaptic input. Figure 1H is a concrete illustration of Theorem 1: it shows that the relative frequency of a specific network state (1,1,1) in a subset of the three neurons 2,7 and 8 converges quickly to its stationary value. Furthermore, it converges to this (same) value regardless of the initial network state (blue/red).

### Stationary distributions of trajectories of network states

Theorem 1 also applies to networks which generate stereotypical trajectories of network activity [41]. For such networks it may be of interest to consider not only the distribution of network states in a short window (e.g. simple states with 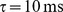, or 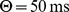), but also the distribution of longer trajectories produced by the network. Indeed, since Theorem 1 holds for Markov states 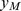 with any fixed window length 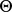, it also holds for values of 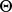 that are in the range of experimentally observed trajectories of network states [41], [49], [50]. Hence, a generic neural circuit 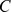 automatically has a unique stationary distribution over *trajectories* of (simple) network states for any fixed trajectory length 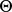. Note that this implies that a neural circuit 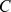 has simultaneously stationary distributions of trajectories of (simple) network states of various lengths for arbitrarily large 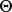, and a stationary distribution of simple network states. This fact is not surprising if one takes into consideration that if a circuit 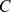 has a stationary distribution over simple network states this does *not* imply that subsequent simple network states represent independent drawings from this stationary distribution. Hence the circuit 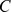 may very well produce stereotypical trajectories of simple network states. This feature becomes even more prominent if the underlying dynamics (the Markov process) of the neural circuit is non-reversible on several time scales.

### Extracting knowledge from internally stored distributions of network states

We address two basic types of knowledge extraction from a stationary distribution 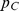 of a network 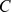: the computation of *marginal probabilities* and *maximal a posteriori (MAP) assignments*. Both computations constitute basic inference problems commonly appearing in real-world applications [51], which are in general difficult to solve as they involve large sums, integrals, or maximization steps over a state space which grows exponentially in the number of random variables. However, already [21], [25] noted that the *estimation* of marginal probabilities would become straightforward if distributions were represented in the brain in a sample-based manner (such that each network state at time 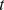 represents one sample from the distribution). Theorem 1 provides a theoretical foundation for how such a representation could emerge in realistic data-based microcircuit models on the implementation level: Once the network 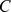 has converged to its stationary distribution, the network state at any time 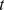 represents a sample from 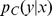 (although subsequent samples are generally not independent). Simultaneously, the subnetwork state 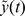 of any subset of neurons represents a sample from the marginal distribution 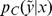. This is particularly relevant if one interprets 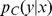 in a given cortical microcircuit 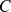 as the posterior distribution of an implicit generative model, as suggested for example by [20] or [21], [22].

In order to place the estimation of marginals into a biologically relevant context, assume that a particular component 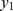 of the network state 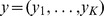 has a behavioral relevance. This variable 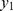, represented by some neuron 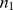, could represent for example the perception of a particular visual object (if neuron 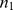 is located in inferior temporal cortex [52]), or the intention to make a saccade into a specific part of the visual field (if neuron 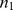 is located in area LIP [53]). Then the computation of the marginal
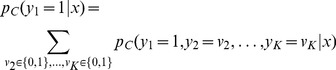
(1)would be of behavioral significance. Note that this computation integrates information from the internally stored knowledge 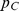 with evidence about a current situation 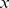. In general this computation is demanding as it involves a sum with exponentially many terms in the network size 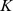.

But according to Theorem 1, the correct marginal distribution 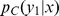 is automatically embodied by the activity of neuron 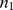. Hence the marginal probability 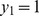 can be estimated by simply observing what fraction of time the neuron spends in the state 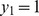, while ignoring the activity of the remaining network [21]. In principle, a downstream neuron could gather this information by integrating the spike output of 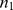 over time.

Marginal probabilities of subpopulations, for example 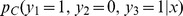, can be estimated in a similar manner by keeping track of how much time the subnetwork spends in the state (1,0,1), while ignoring the activity of the remaining neurons. A downstream network could gather this information, for example, by integrating over the output of a readout neuron which is tuned to detect the desired target pattern (1,0,1).

Notably, the estimation of marginals sketched above is guaranteed by ergodic theory to converge to the correct probability as observation time increases (due to Theorem 1 which ensures that the network is an ergodic Markov process, see Methods). In particular, this holds true even for networks with prominent sequential dynamics featuring, for example, stereotypical trajectories. However, note that the observation time required to obtain an accurate estimate may be longer when trajectories are present since subsequent samples gathered from such a network will likely exhibit stronger dependencies than in networks lacking sequential activity patterns. In a practical readout implementation where recent events might be weighed preferentially this could result in more noisy estimates.

Approximate maximal a posteriori (MAP) assignments to small subsets of variables 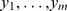 can also be obtained in a quite straightforward manner. For given external inputs 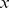, the marginal MAP assignment to the subset of variables 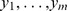 (with some 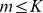) is defined as the set of values 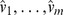 that maximize
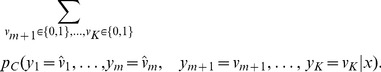
(2)


A sample-based approximation of this operation can be implemented by keeping track of which network states in the subnetwork 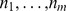 occur most often. This could, for example, be realized by a readout network in a two stage process: first the marginal probabilities 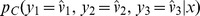 of all 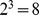 subnetwork states 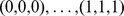 are estimated (by 8 readout neurons dedicated to that purpose), followed by the selection of the neuron with maximal probability. The selection of the maximum could be achieved in a neural network, for example, through competitive inhibition. Such competitive inhibition would ideally lead to a winner-take-all function such that the neuron with the strongest stimulation (representing the variable assignment with the largest probability) dominates and suppresses all other readout neurons.

### Estimates of the required computation time

Whereas many types of computations (for example probabilistic inference via the junction tree algorithm [51]) require a certain computation time, probabilistic inference via sampling from an embodied distribution 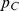 belongs to the class of *anytime computing* methods, where rough estimates of the result of a computation become almost immediately available, and are automatically improved when there is more time for a decision. A main component of the convergence time to a reliable result arises from the time which the distribution of network states needs to become independent of its initial state 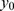. It is well known that both, network states of neurons in the cortex [54] and quick decisions of an organism, are influenced for a short time by this initial state 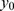 (and this temporary dependence on the initial state 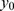 may in fact have some behavioral advantage, since 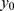 may contain information about preceding network inputs, expectations, etc.). But it has remained unknown, what range of convergence speeds for inference from 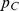 is produced by common models for cortical microcircuits 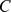.

**Figure 2 pcbi-1003311-g002:**
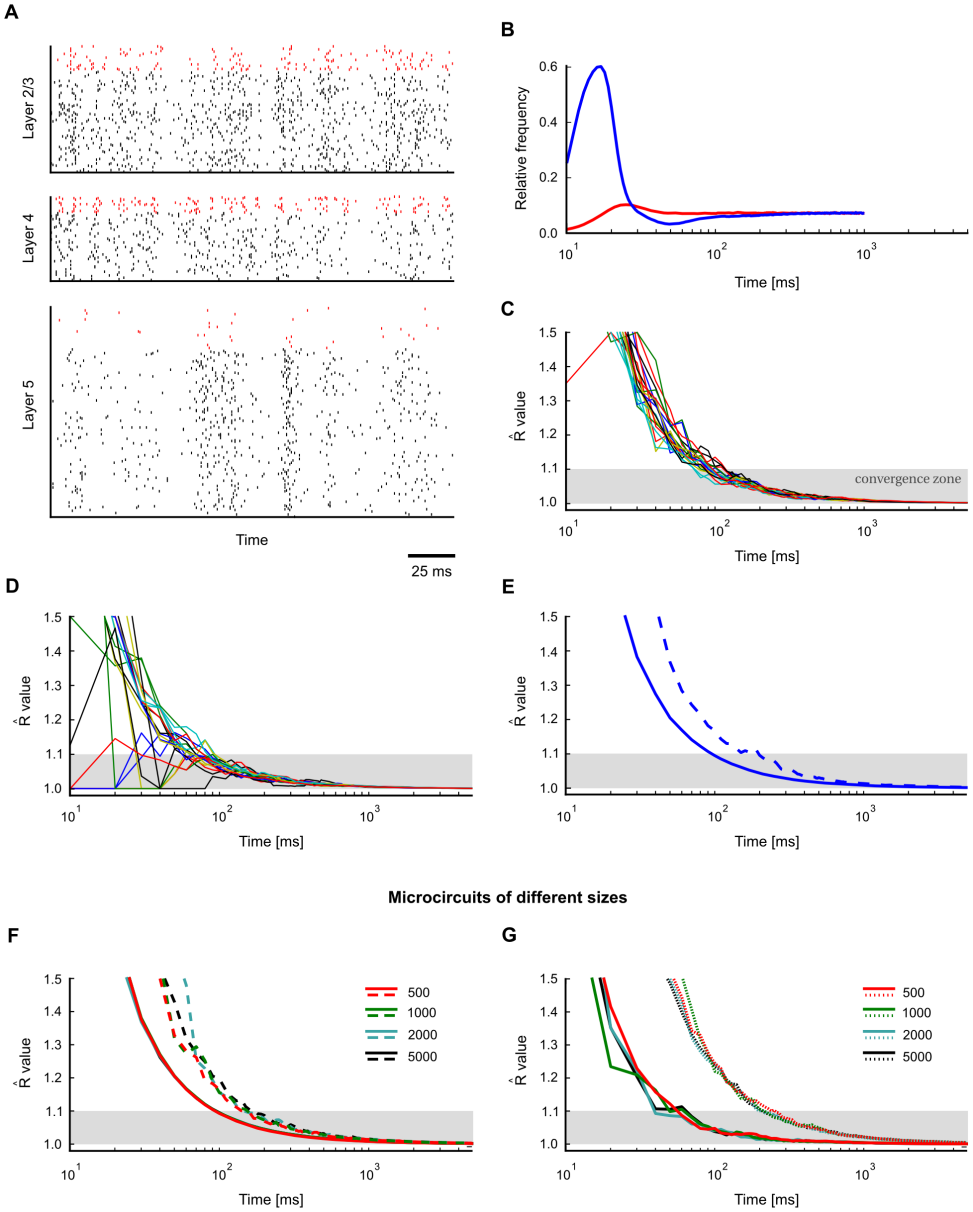
Fast convergence of marginals of single neurons and more complex quantities in a cortical microcircuit model. **A**. Typical spike response of the microcircuit model based on [30] comprising 560 stochastic point neurons. Spikes of inhibitory neurons are indicated in red. **B**. Fast convergence of a marginal for a representative layer 5 neuron (frequency of “on”-state, with 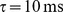) to its stationary value, shown for two different initial Markov states (blue/red). Statistics were obtained for each initial state from 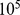 trials. **C**. Gelman-Rubin convergence diagnostic was applied to the marginals of all single neurons (simple states, 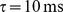). In all neurons the Gelman-Rubin value 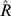 drops to a value close to 1 within a few 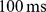, suggesting generally fast convergence of single neuron marginals (shown are 20 randomly chosen neurons; see panel E for a summary of all neurons). The shaded area below 1.1 indicates a range where one commonly assumes that convergence has taken place. **D**. Convergence speed of pairwise spike coincidences (simple states (1,1) of two neurons, 20 randomly chosen pairs of neurons) is comparable to marginal convergence. **E**. Summary of marginal convergence analysis for single neurons in C: Mean (solid) and worst (dashed line) marginal convergence of all 560 neurons. Mean/worst convergence is reached after a few 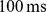. **F**. Convergence analysis was applied to networks of different sizes (500–5000 neurons). Mean and worst marginal convergence of single neurons are hardly affected by network size. **G**. Convergence properties of populations of neurons. Dotted: multivariate Gelman-Rubin analysis was applied to a subpopulation of 30 neurons (5 neurons were chosen randomly from each pool). Solid: convergence of a “random readout” neuron which receives spike inputs from 500 randomly chosen neurons in the microcircuit. It turns out that the convergence speed of such a generic readout neuron is even slightly faster than for neurons within the microcircuit (compare with panel E). A remarkable finding is that in all these cases the network size does not affect convergence speed.

We address this question by analyzing the convergence speed of stochastic computations in the cortical microcircuit model of [30]. A typical network response of an instance of the cortical microcircuit model comprising 560 neurons as in [30] is shown in Figure 2A. We first checked how fast marginal probabilities for single neurons converge to stationary values from different initial network Markov states. We applied the same analysis as in Figure 1H to the simple state (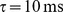) of a single representative neuron from layer 5. Figure 2B shows quite fast convergence of the “on”-state probability of the neuron to its stationary value from two different initial states. Note that this straightforward method of checking convergence is rather inefficient, as it requires the repetition of a large number of trials for each initial state. In addition it is not suitable for analyzing convergence to marginals for subpopulations of neurons (see Figure 2G).

Various more efficient *convergence diagnostics* have been proposed in the context of discrete-time Markov Chain Monte Carlo theory [55]–[58]. In the following, we have adopted the Gelman and Rubin diagnostic, one of the standard methods in applications of MCMC sampling [55]. The Gelman Rubin convergence diagnostic is based on the comparison of many runs of a Markov chain when started from different randomly drawn initial states. In particular, one compares the typical variance of state distributions during the time interval 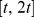 within a single run (within-variance) to the variance during the interval 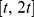 between different runs (between-variance). When the ratio 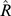 of between- and within-variance approaches 1 this is indicative of convergence. A comparison of panels B and C of Figure 2 shows that in the case of marginals for single neurons this interpretation fits very well to the empirically observed convergence speed for two different initial conditions. Various values between 1.02 [58] and 1.2 [57], [59], [60] have been proposed in the literature as thresholds below which the ratio 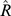 signals that convergence has taken place. The shaded region in Figure 2C–G corresponds to 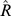 values below a threshold of 1.1. An obvious advantage of the Gelman-Rubin diagnostic, compared with a straightforward empirical evaluation of convergence properties as in Figure 2B, is its substantially larger computational efficiency and the larger number of initial states that it takes into account. For the case of multivariate marginals (see Figure 2G), a straightforward empirical evaluation of convergence is not even feasible, since relative frequencies of 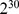 states would have to be analyzed.

Using the Gelman-Rubin diagnostic, we estimated convergence speed for marginals of single neurons (see Figure 2C, mean/worst in Figure 2E), and for the product of the simple states of two neurons (i.e., pairwise spike coincidences) in Figure 2D. We found that in all cases the Gelman-Rubin value drops close to 1 within just a few 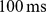. More precisely, for a typical threshold of 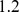 convergence times are slightly below 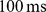 in Figure 2C–E. A very conservative threshold of 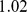 yields convergence times close to 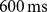.

The above simulations were performed in a circuit of 560 neurons, but eventually one is interested in the properties of much larger circuits. Hence, a crucial question is how the convergence properties scale with the network size. To this end, we compared convergence in the cortical microcircuit model of [30] for four different sizes (500, 1000, 2000 and 5000). To ensure that overall activity characteristics are maintained across different sizes, we adopted the approach of [30] and scaled recurrent postsynaptic potential (PSP) amplitudes inversely proportional to network size. A comparison of mean (solid line) and worst (dashed line) marginal convergence for networks of different sizes is shown in Figure 2F. Notably we find that the network size has virtually no effect on convergence speed. This suggests that, at least within the scope of the laminar microcircuit model of [30], even very large cortical networks may support fast extraction of knowledge (in particular marginals) from their stationary distributions 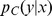.

In order to estimate the required computation time associated with the estimation of marginal probabilities and MAP solutions on small *subpopulations* 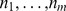, one needs to know how fast the marginal probabilities of *vector-valued* states 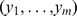 of subnetworks of 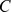 become independent from the initial state of the network. To estimate convergence speed in small subnetworks, we applied a multivariate version of the Gelman-Rubin method to vector-valued simple states of subnetworks (Figure 2G, dotted lines, evaluated for varying circuit sizes from 500 to 5000 neurons). We find that multivariate convergence of state frequencies for a population of 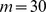 neurons is only slightly slower than for uni-variate marginals. To complement this analysis, we also investigated convergence properties of a “random readout” neuron which integrates inputs from many neurons in a subnetwork. It is interesting to note that the convergence speed of such a readout neuron, which receives randomized connections from a randomly chosen subset of 500 neurons, is comparable to that of single marginals (Figure 2F, solid lines), and in fact slightly faster.

### Impact of different dynamic regimes on the convergence time

**Figure 3 pcbi-1003311-g003:**
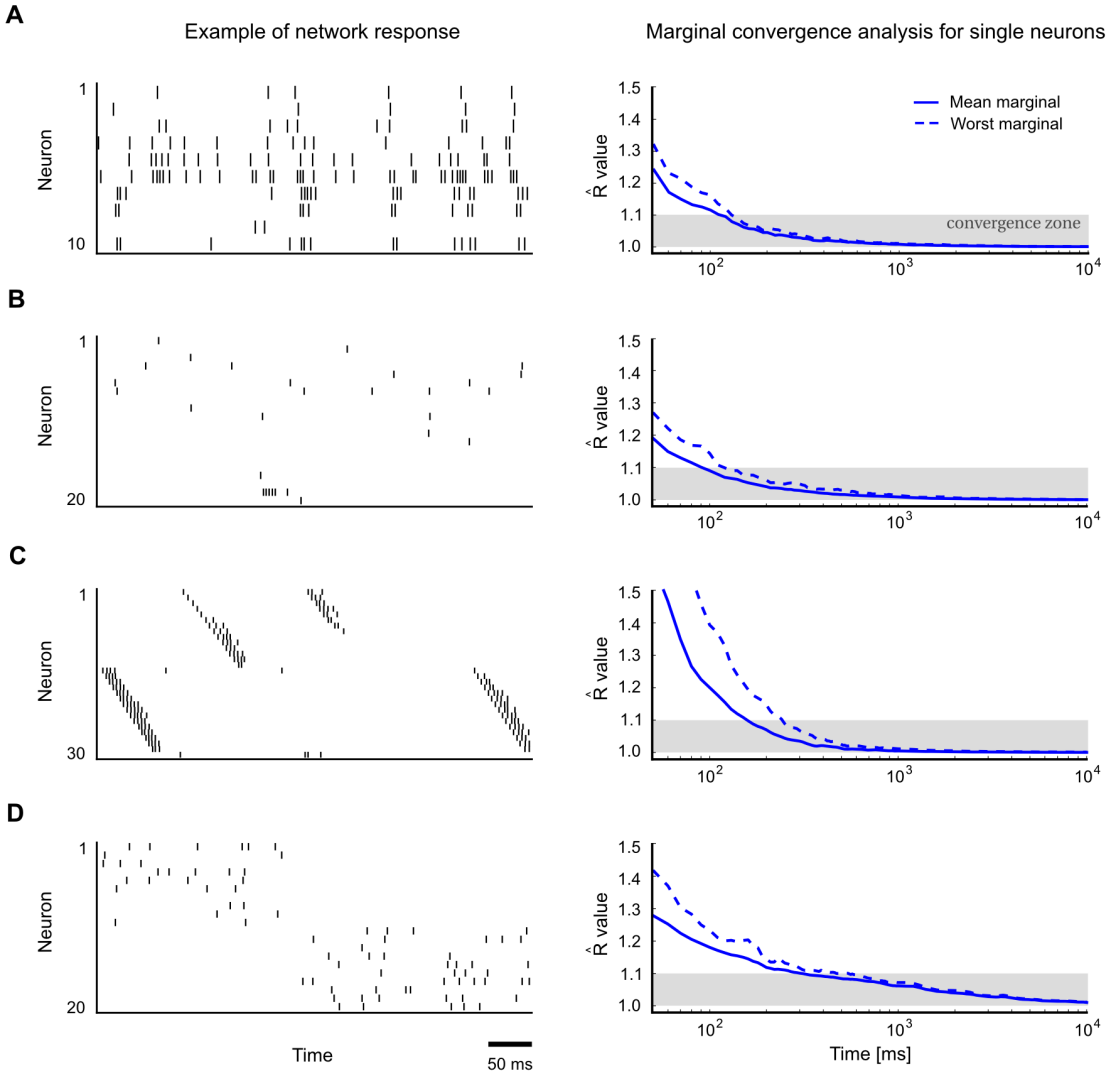
Impact of network architecture and network dynamics on convergence speed. Convergence properties for single neurons (as in Figure 2C) in different network architectures were assessed using univariate Gelman-Rubin analysis. Typical network activity is shown on the left, convergence speed on the right (solid: mean marginal, dashed: worst marginal). **A**. Small cortical column model from Figure 1 (input neurons not shown). **B**. Network with sparse activity (20 neurons). **C**. Network with stereotypical trajectories (50 neurons, inhibitory neurons not shown). Despite strongly irreversible dynamics, convergence is only slightly slower. **D**. Network with bistable dynamics (two competing populations, each comprising 10 neurons). Convergence is slower in this circuit due to low-frequency switching dynamics between two attractors.

An interesting research question is which dynamic or structural properties of a cortical microcircuit model 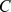 have a strong impact on its convergence speed to the stationary distribution 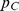. Unfortunately, a comprehensive treatment of this question is beyond the scope of this paper, since virtually any aspect of circuit dynamics could be investigated in this context. Even if one focuses on a single aspect, the impact of one circuit feature is likely to depend on the presence of other features (and probably also on the properties of the input). Nonetheless, to lay a foundation for further investigation, first empirical results are given in Figure 3.

As a reference point, Figure 3A shows a typical activity pattern and convergence speed of single marginals in the small cortical microcircuit model from Figure 1. To test whether the overall activity of a network has an obvious impact on convergence speed, we constructed a small network of 20 neurons (10 excitatory, 10 inhibitory) and tuned connection weights to achieve sparse overall activity (Figure 3B). A comparison of panels A and B suggests that overall network activity has no significant impact on convergence speed. To test whether the presence of stereotypical trajectories of network states (similar to [41]) has a noticeable influence on convergence, we constructed a small network exhibiting strong sequential activity patterns (see Figure 3C). We find that convergence speed is hardly affected, except for the first 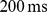 (see Figure 3C). Within the scope of this first empirical investigation, we were only able to produce a significant slow-down of the convergence speed by building a network that alternated between two attractors (Figure 3D).

### Distributions of network states in the presence of periodic network input

In Theorem 1 we had already addressed one important case where the network 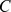 receives dynamic external inputs: the case when external input is generated by some Markov process. But many networks of neurons in the brain are also subject to more or less pronounced periodic inputs (“brain rhythms” [61]–[63]), and it is known that these interact with knowledge represented in distributions of network states in specific ways. For instance, it had been shown in [35] that the phase of the firing of place cells in the hippocampus of rats relative to an underlying theta-rhythm is related to the expected time when the corresponding location will be reached. Inhibitory neurons in hippocampus have also been reported to fire preferentially at specific phases of the theta cycle (see e.g. Figure S5 in [12]). Moreover it was shown that different items that are held in working memory are preferentially encoded by neurons that fire at different phases of an underlying gamma-oscillation in the monkey prefrontal cortex [64] (see [65] for further evidence that such oscillations are behaviorally relevant). Phase coding was also reported in superior temporal sulcus during category representation [66]. The following result provides a theoretical foundation for such phase-specific encoding of knowledge within a framework of stochastic computation in networks of spiking neurons.

#### Theorem 2 (Phase-specific distributions of network states)

*Let* 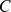 *be an arbitrary model for a network of stochastic spiking neurons as in Theorem 1. Assume now that the vector of input rates* 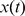 *has in addition to fixed components also some components that are periodic with a period* 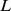 *(such that each input neuron* 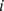 *emits a Poisson spike train with an* 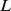 *-periodically varying firing rate* 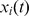 *). Then the distribution of network states* 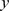 *converges for every phase* 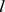 *(* 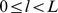 *) exponentially fast to a unique stationary distribution of network states* 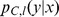 *at this phase* 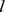 *of the periodic network input* 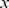 *.*

Hence, a circuit 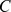 can potentially store in each clearly separable phase 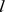 of an (externally) imposed oscillation a different, phase-specific, stationary distribution 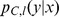. Below we will address basic implications of this result in the context of two experimentally observed phenomena: stereotypical trajectories of network states and bi-stable (or multi-stable) network activity.

**Figure 4:**
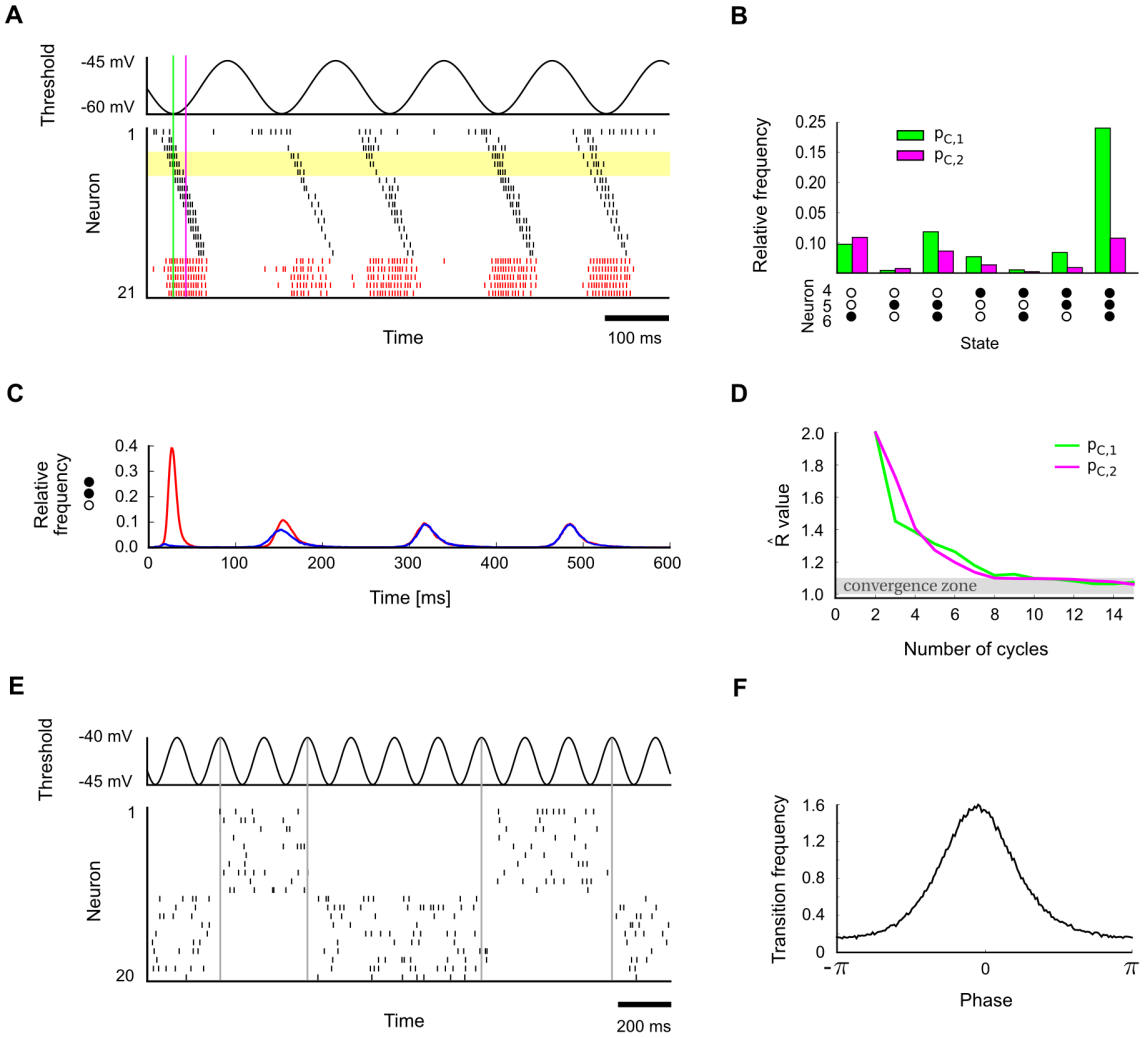
Emergence of phase-specific stationary distributions of network states in the presence of periodic network input. **A**. A network with a built-in stereotypical trajectory is stimulated with a 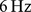 background oscillation. The oscillation (top) is imposed on the neuronal thresholds of all neurons. The trajectories produced by the network (bottom) become automatically synchronized to the background rhythm. The yellow shading marks the three neurons for which the analysis in panels B and C was carried out. The two indicated time points (green and purple lines) mark the two phases for which the phase-specific stationary distributions are considered in panels B and D (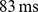 and 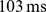 into the cycle, with phase-specific distributions 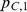 and 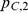, respectively). **B**. The empirically measured distributions of network states are observed to differ significantly at two different phases of the oscillation (phases marked in panel A). Shown is for each phase the phase-specific marginal distribution over 3 neurons (4, 5 and 6), using simple states with 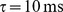. The zero state (0,0,0) is not shown. The empirical distribution for each phase 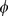 was obtained from a single long run, by taking into account the network states at times 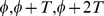, etc., with cycle length 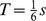. **C**. Illustration of convergence to phase-specific stationary distributions. Shown is the relative frequency of subnetwork state (1,1,0) on the subset of neurons 4,5 and 6 over time, when the network is started from two different initial states (red/blue). In each case, the state frequency quickly approaches a periodic limit cycle. **D**. Convergence to phase-specific stationary distributions takes place within a few cycles of the underlying oscillation. Shown is the multivariate Gelman-Rubin convergence analysis to the phase-specific stationary distribution for two different phases. **E**. Bi-stable network under the influence of a 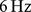 background oscillation. **F**. In response to the periodic stimulation, transitions between the two attractors (modes) become concentrated around a specific phase of the distribution.

Figure 4A–D demonstrates the emergence of phase-specific distributions in a small circuit (the same as in Figure 3C but with only one chain) with a built-in stereotypical trajectory similar to a spatial path sequence generated by hippocampal place cell assemblies [35], [36]. Figure 4A shows a typical spike pattern in response to rhythmic background stimulation (spikes from inhibitory neurons in red). The background oscillation was implemented here for simplicity via direct rhythmic modulation of the spiking threshold of all neurons. Note that the trajectory becomes particularly often initiated at a specific phase of the rhythm (when neuronal thresholds are lowest), like in experimental data 35,36. As a result, different phases within a cycle of the rhythm become automatically associated with distinct segments of the trajectory. One can measure and visualize this effect by comparing the frequency of network states which occur at two different phases, i.e., by comparing the stationary distributions 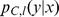 for these two phases. Figure 4B shows a comparison of phase-specific marginal distributions on a small subnetwork of 3 neurons, demonstrating that phase-specific stationary distributions may indeed vary considerably across different phases. Convergence to the phase-specific stationary distributions 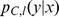 can be understood as the convergence of the probability of any given state to a periodic limit cycle as a function of the phase 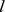 (illustrated in Figure 4C). An application of the Gelman-Rubin multivariate diagnostic suggests that this convergence takes places within a few cycles of the theta oscillation (Figure 4D).

Theta-paced spatial path sequences in hippocampus constitute a particularly well-studied example of phase-specific network activity [35]. Our theoretical framework suggests a novel interpretation of these patterns as samples from a Markov chain with a phase-dependent stationary distribution of network states induced by the theta-rhythm. A basic prediction of this interpretation is that two trajectories in successive theta cycles should exhibit significantly stronger similarities than two trajectories from randomly chosen cycles (due to inherent temporal dependencies of the Markov chain). Two trajectories from distant cycles, on the other hand, should relate to each other similarly as randomly chosen pairs of trajectories. Evidence for such an effect has been reported recently by [36], where it was found that “sequences separated by 20 cycles approach random chance, whereas sequences separated by only a single theta cycle are more likely to be similar to each other.”

The previously described theoretical framework also provides an interesting new perspective on multi-stability, a wide-spread phenomenon which has been observed in various sensory domains [67], [68]. Different authors have noted that multi-stability, both on the neuronal and perceptual level, could be understood as a side effect of sampling from a multi-modal distribution [19], [21], [69]. Recent data from hippocampus suggest that oscillations, which had previously received little attention in this context, may play an important role here: [37] found that switching between different attractors ( = modes of the stationary distribution in our terminology) occurs preferentially at a specific phase during the theta cycle, whereas activity patterns within each cycle preferentially stayed in one attractor. Hence, the precise timing of switching between modes was found to be strongly tied to the theta rhythm. Such chunking of information in separate packages (theta cycles) has been proposed as an important constituent of neural syntax [42].

In Figure 4E we reproduce phase-dependent switching in a simple network model of bi-stable dynamics (the same network as in Figure 3D) in the presence of a 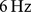 background oscillation. Indeed, we find that switching occurs preferentially at a specific phase of the oscillation (see Figure 4F) when the total firing rate of the network is lowest. Note that this is consistent with [37] who found that the separation between representations in different cycles was strongest at the point of the lowest average firing rate in the population (see Figure 1b in [37]). This phenomenon can be explained in our model by noting that the attractors are deeper during periods of high network activity. Conversely, attractors are more shallow when the population firing rate is lower, leading to an increased transition probability between attractors. If one takes a closer look at Proposition 1 and Lemma 1 in Methods one sees that this is also consistent with our theoretical framework: A lower population firing rate 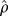 translates into a smaller contraction factor 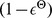, implying a tighter bound on the contraction speed of state distributions and thus higher transition probabilities to radically different states from the current (initial) network state.

Altogether, one sees that the presence of background oscillations has relevant functional implications on multi-stability. In particular, the presence of background oscillations in multi-stable networks facilitates both exploitation within a cycle and exploration across cycles: Within a cycle high firing rates force the network into one of the attractors, thereby avoiding interference with other attractors and facilitating the readout of a consistent network state. At the end of a cycle low firing rates allow the network to switch to different attractors, thereby promoting fast convergence to the stationary distribution. The rhythmic deepening and flattening of attractors and the resulting phase-specific attractor dynamics could be particularly useful for the extraction of information from the circuit if downstream networks are phase-locked to the same rhythm, as reported, for example, for the interactions between neurons in hippocampus and prefrontal cortex [70].

### Solving constraint satisfaction problems in networks of spiking neurons

Whenever an inhibitory neuron fires, it reduces for a short while the probability of firing for its postsynaptic targets. In fact, new experimental data [71] show that inhibitory neurons impose quite powerful constraints on pyramidal cells. But also how pyramidal cells are embedded into their network environment imposes constraints on local network activity. From this perspective, the resulting firing patterns of a cortical microcircuit can be viewed as stochastically generated solutions of an immensely complex constraint satisfaction problem, that is defined both by external inputs 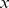 to the circuit and by the way each excitatory and inhibitory neuron is embedded into its circuit environment. Constraint satisfaction problems are from the computational perspective a particularly interesting class of problems, because many tasks that a brain has to solve, from the generation of a percept from unreliable and ambiguous sources to higher level tasks such as memory recall, prediction, planning, problem solving, and imagination, can be formulated as constraint satisfaction problems [72]. However, numerous constraint satisfaction problems are known to be NP-hard, thereby limiting the applicability of exact solution strategies. Instead, approximate or heuristic algorithms are commonly used in practice (for example evolutionary algorithms [73]). Here we propose that networks 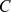 of spiking neurons with noise have an inherent capability to solve constraint satisfaction problems in an approximate (heuristic) manner through their stochastic dynamics. The key principle is that those network states 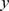, which satisfy the largest number of local constraints, have the highest probability under the distribution 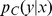. These constraints are imposed by the way each neuron of 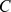 is embedded into the circuit, and the current external input 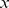 which can selectively activate or deactivate specific constraints.

**Figure 5:**
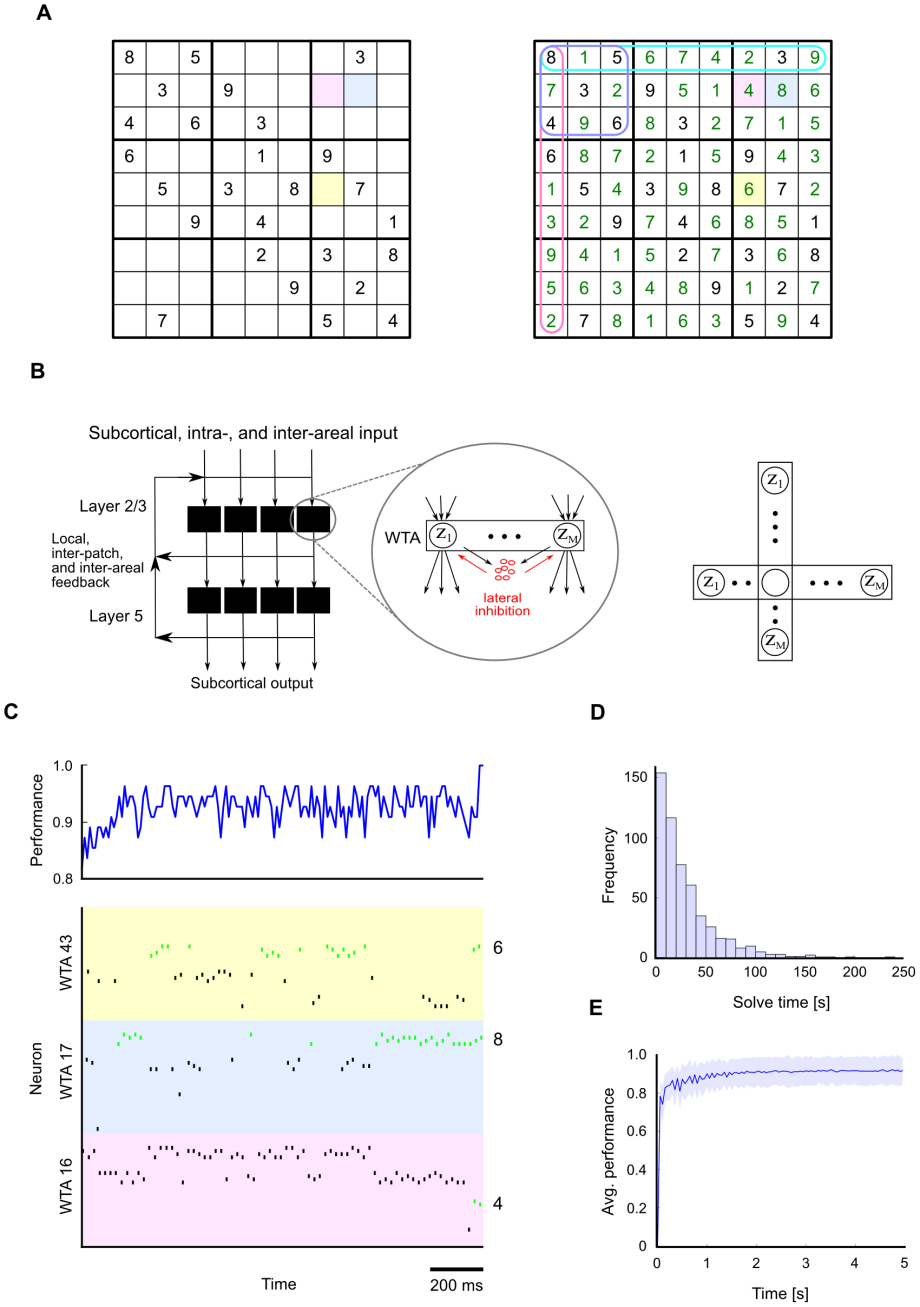
Solving Sudoku, a constraint satisfaction problem, through structured interactions between stochastically firing excitatory and inhibitory neurons. **A**. A “hard” Sudoku puzzle with 26 given numbers (left). The solution (right) is defined uniquely by the set of givens and the additional constraints that each digit must appear only once in each row, column and 3×3 subgrid. **B**. An implementation of the constraints of the Sudoku game in a spiking neural network 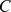 consists of overlapping WTA circuits. WTA circuits are ubiquitous connection motifs in cortical circuits [29]. A WTA circuit can be modeled by a set of 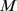 stochastically spiking output neurons 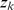 that are subject to lateral inhibition (left). The same pyramidal cell can be part of several such WTA motifs (right). In the Sudoku example, each digit in a Sudoku field is associated with four pyramidal cells which vote for this digit when they emit a spike. Each such pyramidal cell participates in four WTA motifs, corresponding to the constraints that only one digit can be active in each Sudoku field, and that a digit can appear only once in each row, column and 3×3 subgrid. **C**. A typical network run is shown during the last 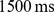 before the correct solution was found to the Sudoku from panel A (the total solve time was approximately 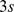 in this run, see panel D for statistics of solve times). The network performance (fraction of cells with correct values) over time is shown at the top. The spiking activity is shown for 3 (out of the 81) WTA motifs associated with the 3 colored Sudoku fields in A and B. In each of these WTA motifs there are 36 pyramidal cells (9 digits and 4 pyramidal cells for each digit). Spikes are colored green for those neurons which code for the correct digit in each Sudoku field (6, 8 and 4 in the example). **D**. Histogram of solve times (the first time the correct solution was found) for the Sudoku from panel A. Statistics were obtained from 1000 independent runs. The sample mean is 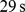. **E**. Average network performance for this Sudoku converges quickly during the first five seconds to a value of 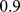, corresponding to 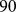
% correctly found digits (average taken over 1000 runs; shaded area: 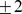 standard deviations). Thereafter, from all possible 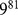 configurations the network spends most time in good approximate solutions. The correct solution occurs particularly often, on average approximately 2% of the time (not shown).

We have selected a specific constraint satisfaction problem for demonstrating the capability of networks of spiking neurons to generate rapidly approximate solutions to constraint satisfaction problems through their inherent stochastic dynamics: solving Sudoku puzzles (see Figure 5A). Sudoku is a well-suited example because it is complex enough to be representative for many problem solving tasks, and lends itself well to visual interpretation and presentation (but note that we do not aim to model here how humans solve Sudoku puzzles). The rules of the Sudoku game can be easily embedded into common models for cortical microcircuits as recurrent networks of Winner-Take-All (WTA) microcircuit motifs [29]. Each WTA motif is an ensemble of pyramidal cells (on layers 2/3 or 5/6) that are subject to lateral inhibition (see Figure 5B). Each pyramidal cell can in fact be part of several interlocking WTA motifs (Figure 5B, right).

This architecture makes it easy to impose the interlocking constraints of Sudoku (and of many other constraint satisfaction problems). Each pyramidal cell (or each local group of pyramidal cells) votes for placing a particular digit into an empty field of the grid, that is not dictated by the external input 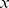. But this pyramidal cell is subject to the constraints that only one digit can be placed into this field, and that each digit 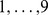 occurs only once in each column, in each row, and in each 3×3 sub-grid. Hence each pyramidal cell is simultaneously part of four inhibitory subnetworks (WTA motifs).

A specific puzzle can be entered by providing strong input 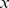 to those neurons which represent the given numbers in a Sudoku (Figure 5A, left). This initiates a quite intuitive dynamics: “Clamped” neurons start firing strongly, and as a consequence, neurons which code for conflicting digits in the same Sudoku field, the same row, column or 3×3 sub-grid, become strongly inhibited through di-synaptic inhibition. In many Sudoku fields this will lead to the inhibition of a large number of otherwise freely competing neurons, thereby greatly reducing the space of configurations generated by the network. In some cases, inhibition will immediately quieten all neurons except those associated with a single remaining digit (the only choice consistent with the givens). In the absence of competition, these uninhibited neurons will start firing along with the givens, thereby further constraining neighboring neurons. This form of inhibitory interaction therefore implicitly implements a standard strategy for solving easy Sudokus: checking for fields in which only one possibility remains. In harder Sudokus, however, this simple strategy alone would be typically insufficient, for example when several possibilities remain in all fields. In such cases, where inhibition leaves more than one possible digit open, a tentative digit will be automatically picked randomly by those neurons which happen to fire first among its competitors. This ensures that, instead of getting stuck, the network automatically explores potential configurations in situations where multiple possibilities remain. Altogether, through this combination of constraint enforcement and random exploration, those network states which violate few constraints (good approximate solutions) are visited with much higher probability than states with conflicting configurations. Hence, most time is spent in good approximate solutions. Furthermore, from all 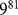 Sudoku configurations the solving configuration is visited in this process especially often.

Figure 5C shows a typical network run during the last 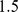 seconds (out of a total simulation time of approximately 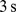) before the correct solution was found to the Sudoku puzzle from Figure 5A. For this simulation we modeled lateral inhibition in each WTA motif by reciprocally connecting each neuron in the subnetwork to a single inhibitory neuron. For each of the 9 digits in a Sudoku field, we created an associated local group of four pyramidal cells. This can be seen in Figure 5C, where spike responses of pyramidal cells associated with three different Sudoku fields are shown (the three colored fields in Figure 5A and B). Each field has 9 possible digits, and each digit has four associated neurons. Hence, for each of the three Sudoku fields (WTA motifs), 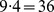 neurons are shown. Spikes are colored black for those neurons which code for a wrong digit, and green for the four neurons which code for the correct digit in a Sudoku field (the correct digits in Figure 5C are 6, 8 and 4). The overall performance of the network (fraction of correctly solved fields) during the last 1.5 seconds before the solution is found is shown in Figure 5C above.

In our simulations we found that the solve time (the time until the correct solution is found for the first time) generally depends on the hardness of the Sudoku, in particular on the number of givens. For the “hard” Sudoku with 26 givens from Figure 5A, solve times are approximately exponentially distributed at an average of 29 seconds (Figure 5D). The average performance during the first five seconds of a run (obtained from 1000 independent runs) is shown in Figure 5E. The plot shows quick convergence to a (stationary) average performance of approximately 0.9. This demonstrates that the network spends on average most time in approximate solutions with high performance. Among these high-performance solutions, the correct solution occurs especially often (on average 2% of the time).

## Discussion

### A theoretical foundation for memory-based stochastic computation in cortical microcircuits

We have shown that for common noise models in cortical microcircuits, even circuits 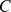 with very detailed and diverse non-linear neurons and synapses converge exponentially fast to a stationary distribution 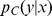 of network states 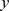. This holds both for external inputs 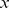 that consist of Poisson spike trains of a fixed rate, and for the case where 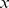 is periodic, or generated by some Markov process with a stationary distribution. The same mathematical framework also guarantees exponentially fast convergence to a stationary distribution of *trajectories* of network states (of any fixed time length), thereby providing a theoretical foundation for understanding stochastic computations with experimentally observed stereotypical trajectories of network states. These results extend and generalize previous work in [17] and [18] in two ways. First, previous convergence proofs had been given only for networks of simplified neurons in which the (sub-threshold) neuronal integration of pre-synaptic spikes was assumed a linear process, thereby excluding the potential effects of dendritic non-linearities or synaptic short-term dynamics. Second, previous work had focused only on the case where input is provided by neurons with fixed firing rates (a special case of Theorem 1). In addition we show that these convergence proofs can be derived from a fundamental property of stochastic spiking networks, that we have formulated as the Contraction Lemma (Lemma 1 in Methods).

The stationary distribution 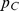 provides an attractive target for investigating the stochastic computing capabilities of data-based models 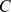 for local circuits or larger networks of neurons in the brain. In contrast to the much simpler case of Boltzmann machines with non-spiking linear neurons and symmetric synaptic connections, it is unlikely that one can attain for cortical microcircuit models 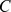 a simple analytical description of 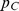. But our computer simulations have shown that this is not necessarily an obstacle for encoding salient constraints for problem solving in 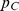, and for merging knowledge that is encoded in 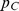 with online information from external inputs 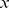 in quite fast stochastic computations. In fact, the resulting paradigm for computations in cortical microcircuits supports anytime computing, where one has no fixed computation time. Instead, first estimates of computational results can be produced almost immediately, and can be rapidly communicated to other circuits. In this way, no processor (circuit) has to idle until other processors have completed their subcomputations, thereby avoiding the arguably most critical general bottleneck of massively parallel computing systems. Instead, each microcircuit 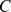 can contribute continuously to an iterative refinement of a global computation.

### Estimates for the computation time of stochastic computations

Our computer simulations for a standard cortical microcircuit model 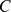 suggest that convergence to 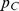 is fast enough to support knowledge extraction from this distribution 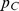 within a few 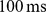, i.e. within the typical computation time of higher-level brain computations. These first estimates need to be corroborated by further theoretical work and computer simulations. In particular, the relationship between the structure and dynamics of cortical microcircuits and their convergence speed merits further investigation. Furthermore, in the case where 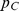 is a multi-modal distribution there exists an obvious tradeoff between the convergence speed to 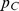 and the typical duration of staying in an “attractor” (i.e., a region of the state space which has high probability under 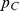). Staying longer in an attractor obviously facilitates the readout of the result of a computation by downstream networks. A number of experimental data suggest that neuromodulators can move neural circuits (at least in the prefrontal cortex) to different points on this tradeoff curve. For example it is argued in [74], [75] that the activation of 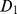 receptors through dopamine deepens all basins of attraction, making it harder for the network state to leave an attractor. Additional molecular mechanisms that shift the tradeoff between fast sampling (exploration) and the temporal stability of found solutions are reviewed in [76]. Another interesting perspective on convergence speed is that slow convergence may be beneficial for certain computations in specific brain areas (especially early sensory areas). Slow convergence enlarges the time span during which the network can integrate information from non-stationary external inputs [77]–[79]. In addition the initial state 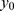 of a network may contain information about preceding events that are computationally useful. Those considerations suggest that there exist systematic differences between the convergence speed to 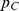 in different neural systems 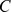, and that it can be modulated in at least some systems 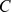 dependent on the type of computational task that needs to be solved.

Another important issue is the tradeoff between sampling time and sampling accuracy. In high-level cognitive tasks, for example, it has been argued that “approximate and quick” sample-based decisions are often better than “accurate but slow” decisions [80], [81]. Of particular interest in this context is the analysis of [81] who studied the time-accuracy tradeoff during decision making, under the assumption that the mind performs inference akin to MCMC sampling. Due to the nature of MCMC sampling, early samples before convergence (during the burn-in period) are biased towards the initial state of the system. In the absence of time pressure, the optimal strategy is therefore to wait and collect samples for a long period of time (in theory indefinitely). In the presence of even moderate time costs, however, the optimal sampling time can be shown to be finite, a result which can provide a rational explanation of the anchoring effect in cognitive science [81] (under time pressure people's decisions are influenced by their “initial state”). Notably, the analysis of [81] was based on the assumption that the MCMC algorithm exhibits geometric convergence, the discrete-time equivalent to the exponential convergence speed proved in this paper for stochastic spiking networks. Applying a similar analysis to study optimal time-accuracy tradeoff points in cortical microcircuits therefore presents a promising avenue for future research.

### Which probability distributions 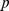 can be encoded as a stationary distribution 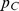 of some neural circuit 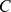
?

It had been shown in [21] and [22] that, under certain assumptions on the neuron models and circuit structure, in principle every joint distribution 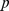 over discrete-valued random variables can be represented as a stationary distribution 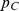 of some network 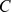 of spiking neurons. Forthcoming unpublished results suggest that such internal representations of a given distribution 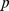 can even be learned from examples drawn from 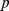. This will provide a first step towards understanding how the stationary distribution 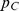 of a microcircuit can be adapted through various plasticity processes to encode salient constraints, successful solution strategies (rules), and other types of knowledge. This research direction promises to become especially interesting if one takes into account that knowledge can not only be encoded in the stationary distribution of network states, but also in the simultaneously existing stationary distribution of trajectories of network states.

### Relationship to attractor networks and transients between attractors

Attractor neural networks [82] were originally deterministic computational models, where gradient descent leads the network from some given initial state 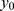 (the input for the computation) to the lowest point of the attractor (the output of the computation) in whose basis of attraction 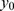 lies. The computational capability of an attractor neural network is substantially larger if its attractor landscape can be reconfigured on the fly by external input 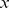, as in [83] and in the Sudoku example of this article. This usually requires that the attractors are not programmed directly into the network parameters, but emerge from some more general computational principles (e.g. constraint satisfaction). Attractor neural networks gain additional computational capability if there is some noise in the system [84]. This enables the network to leave after a while suboptimal solutions [85]. Alternative modeling frameworks for the transient dynamics of neural systems are provided by the liquid computing model [77], and on a more abstract level by sequences of metastable states in dynamical systems [86]. Here we propose to view both transient and attractor dynamics of complex data-based circuits 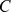 from the perspective of probabilistic inference, in particular as neural sampling [21] (or more abstractly: as MCMC sampling) from their inherent probability distribution 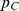 over network states (or trajectories of network states), that serves as the knowledge base of these neural systems.

### A new computational framework for analyzing brain activity

We had focused in our computer simulations on the investigation of the stationary distribution 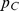 for models 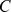 of cortical microcircuits. But the results of Theorem 1 and Theorem 2 are of course much more general, and in principle apply to models 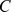 for networks of neurons in the whole brain [87]. This perspective suggests understanding spontaneous brain activity (see [9]) as sampling from this global distribution in the absence of external input, and brain computations with external inputs 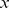 as sampling of brain states from conditional distribution 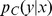, thereby merging the knowledge base 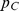 of the brain with incoming new information 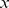. This computational framework could in principle explain how the brain can merge both types of information in such seemingly effortless manner, a capability that can only partially be reproduced in artificial devices with current technology. Large-scale computer simulations will be needed to test the viability of this hypothesis, in particular the relationship between the known global structure of the brain network 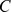 and properties of its stationary distribution 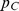, and the convergence speed to 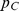. Possibly the brain uses an important trick to speed up convergence during brain-wide sampling, for example by sampling during any concrete brain computation only from a subnetwork 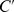 of 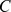: those brain areas that control variables that are relevant for this computation. Functional connectivity would be explained from this perspective as opening of communication channels that support sampling from the (marginal) joint distribution of those variables that are stored within the functionally connected brain areas. Structured spontaneous brain activity [9] would then receive a functional interpretation in terms of updating these marginal joint distributions on the basis of newly acquired knowledge.

### Stochastic solutions of constraint satisfaction problems as a paradigm for higher level brain computation

A surprisingly large number of computational tasks that the brain has to solve, from the formation of a percept from multi-modal ambiguous sensory cues, to prediction, imagination, motor planning, rule learning, problem solving, and memory recall, have the form of constraint satisfaction problems: A global solution is needed that satisfies all or most of a set of soft or hard constraints. However, this characterization per se does not help us to understand how the brain can solve these tasks, because many constraint satisfaction problems are computationally very demanding (in fact, often NP-hard [88]), even for a fast digital computer. In the Sudoku example we have shown that the inherent stochastic dynamics of cortical microcircuits provides a surprisingly simple method for generating *heuristic* solutions to constraint satisfaction problems. This is insofar remarkable, as this computational organization does not require that specific algorithms are programmed into the network for solving specific types of such problems (as it is for example needed for solving Sudoku puzzles according to the ACT-R approach [89]). Rather, it suffices that salient constraints are encoded into the network (e.g. through learning) in such a way that they make certain firing patterns of a subset of neurons more or less likely.

Future work will need to investigate whether and how this approach can be scaled up to larger instances of NP-complete constraint satisfaction problems. For example, it will be interesting to see whether stochastic networks of spiking neurons can also efficiently generate heuristic solutions to energy minimization problems [90] arising in visual processing.

Furthermore, additional research is needed to address suitable readout mechanisms that stabilize and evaluate promising candidate solutions (see [76] for an experimentally supported mechanism that might contribute to this function). This is an important issue since, in its current form, the network will simply continue the stochastic exploration of heuristic solutions even after it has found the optimal solution. Therefore, in the absence of additional mechanisms the network is not able to hold on to (or store) previously found (near-)optimal solutions. To solve this issue one could consider, for example, one or several networks 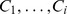 which generate in parallel heuristic solutions to a given problem. The output of these networks could then be further processed and integrated by a readout network 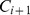 which attempts to extract a MAP solution, for example by adopting a solution from some 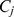 only if it has higher value than the currently stored state. Hence, the sampling networks 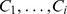 would have stationary distributions 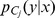 which encourage exploration and broadly assign probability to many different heuristic solutions, whereas the readout network would ideally exhibit a sharply peaked stationary distribution at the global optimum of the constraint satisfaction problem. Studying the feasibility of this approach requires further research.

### Relationship to models for probabilistic inference in cognitive science

A substantial number of behavioral studies in cognitive science (see e.g. [69], [91]–[94]) have arrived at the conclusion that several of the previously discussed higher level mental operations are implemented through probabilistic inference. Some of the underlying data also suggest that probabilistic inference is implemented in the brain through some form of sampling (rather than through arithmetical approaches such as belief propagation [44]). But according to [94]: “The key research questions are as follows: What approximate algorithms does the mind use, how do they relate to engineering approximations in probabilistic AI, and how are they implemented in neural circuits?” This article contributes to these fascinating questions by providing a rigorous theoretical foundation for the hypothesis that neural circuits in the brain represent complex probability distributions 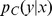 through sampling. In addition, we have provided evidence that this form of sampling in cortical microcircuits may be fast enough to facilitate the approximate estimation of marginals or marginal MAP assignments, which commonly appear in real-world inference tasks, within a few 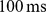. A major challenge for future work will be to understand also neuronal plasticity on the implementation level from this perspective. For example, how can prior knowledge be acquired and integrated into the stationary distribution 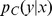 of a realistic circuit 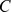 (featuring short-term plasticity, dendritic processing, etc.) in an autonomous fashion, and in a manner consistent with statistically optimal learning [25]?

### Long-term plasticity and other slower features of network dynamics

In biological networks it is reasonable to assume that the network dynamics unfolds on a continuum of time scales from milliseconds to days. Our goal in this article was to focus on stochastic computations on shorter time scales, between a few milliseconds to seconds. To this end we assumed that there exists a clear separation of time scales between fast and slow dynamical network features, thus allowing us to exclude the effect of slower dynamical processes such as long-term plasticity of synaptic weights during these shorter time scales. In network models and experimental setups where slower processes significantly influence (or interfere with) the dynamics on shorter time scales, it would make sense to extend the concept of a stationary distribution to include, for example, also the synaptic parameters as random variables. A first step in this direction has been made for neurons with linear sub-threshold dynamics and discretized synapses in [18].

### Deterministic network models and chaos

Deterministic network models such as leaky integrate-and-fire neurons without noise (no external background noise, no synaptic vesicle noise and no channel noise) violate the assumptions of Theorem 1 and 2. Furthermore, although realistic neurons are known to possess various noise sources, the theoretical assumptions could in principle still fail if the network is not *sufficiently* stochastic: this would happen, for example, if there exists some strong input (within the limits of typical input activity) which entirely overrules the noise, leading to a firing probability 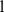 in some time interval 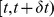 during the network simulation. Such deterministic behavior would correspond to the instantaneous firing rate of a stochastic neuron becoming infinite at some point during that interval (in violation of assumption A2, see Methods: Scope of theoretical results). From an empirical perspective, a simple necessary condition for sufficient stochasticity is the presence of trial-to-trial variability for each single spike produced by a network. Consider, for example, the spike times generated by a specific neuron in a network simulation, in response to some fixed input spike train. If there exists a spike which always occurs at the exact same time during multiple repetitions of this experiment starting from identical initial states, then the assumptions of Theorem 1 and 2 are obviously violated.

For deterministic (or insufficiently stochastic) networks the question arises whether convergence to a unique stationary distribution may still occur under appropriate conditions, perhaps in some modified sense. Notably, it has been recently observed that deterministic networks may indeed lead to apparently stochastic spiking activity [95], [96]. This apparent stochasticity was linked to chaotic spiking dynamics. This suggests that chaos may act as a substitute for “real” noise in deterministic networks (similar to pseudo random-number generators emulating true randomness): Chaotic systems are sensitive to small perturbances in initial conditions, and may thus exponentially amplify otherwise insignificant noise sources such as ubiquitous thermal noise [5]. Thus, chaos could play an important role in both emulating and amplifying stochasticity on the network level.

[96] focused their analysis of stochasticity on firing rate fluctuations and spiking irregularity, and it remains unclear whether these networks would still appear stochastic if one takes into account full network states (as in this article). The Gelman-Rubin convergence analysis of population activity proposed in this paper could be applied to provide some insight into this question. A more thorough investigation of chaos in the context of our results would also call for a rigorous theoretical analysis of ergodic properties of chaotic spiking networks.

### Further experimentally testable predictions

Our theoretical results demonstrate that every neural system 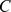 has a stationary distribution 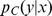 of network states 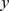. This can be tested experimentally, for various behavioral regimes and external inputs 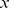. A first step in this direction has already been carried out in [20] (see also the discussion in [97]). The hypothesis that 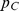 serves (for “neutral” external inputs 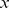) as a prior for probabilistic inference through sampling suggests that 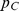 is constantly modified through prior experience (see [98], [99] for first results) and learning (see [10] for fMRI data).

Our Theorem 2 suggests in addition that neural systems 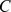 that have a prominent rhythm (such as for example the theta oscillation in the hippocampus) are able to store *several* stationary distributions 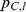 of network states, one for each clearly separable phase 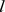 of this rhythm. It has already been shown in a qualitative manner that in some behavioral situations certain states 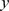 appear with substantially high probability at specific phases 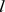 of the rhythm (see e.g. [36], [62], [64], [66], [100]). But a systematic experimental analysis of phase-dependent distributions of network states in the style of [20] is missing.

Our Theorem 1 predicts in addition that a generic neural circuit 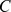 also has a stationary distribution over *trajectories* of network states. The existence of stereotypical trajectories of network states in the awake brain has been frequently reported (see e.g. [40], [41], [98], [101]). But a statistical analysis of the distribution of such trajectories, especially also during spontaneous activity, is missing. Of particular interest is the relationship between the distribution of trajectories and the stationary distribution of (simple) network states. Do some network states 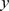 typically have a high probability because they occur in some high probability trajectory? And how does the distribution of trajectories change during learning?

The model for problem solving that we have presented in Figure 5 suggests that external constraints have a significant and characteristic impact on the structure of the stationary distribution 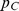, by reducing the probability of network states which are inconsistent with the current constraints 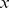. In principle, this could be analyzed experimentally. In addition, this model suggests that there may be special mechanisms that prolong the time span during which a neural system 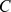 stays in a network state 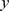 with high probability under 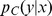, in order to support a readout of 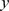 by downstream networks. These mechanisms need to be revealed through experiments.

### New ideas for neuromorphic computation

The Sudoku example has shown that networks of spiking neurons with noise are in principle able to carry out quite complex computations. The constraints of many other demanding constraint satisfaction problems, in fact even of many NP-complete problems, can be encoded quite easily into circuit motifs composed of excitatory and inhibitory spiking neurons, and can be solved through the inherent stochastic dynamics of the network. This provides new computational paradigms and applications for various energy-efficient implementations of networks of spiking neurons in neuromorphic hardware, provided they can be equipped with sufficient amounts of noise. In particular, our results suggest that attractive computational properties of Boltzmann machines can be ported into spike-based hardware. These novel stochastic computing paradigms may also become of interest for other types of innovative computer hardware: Computer technology is approaching during the coming decade the molecular scale, where noise is abundantly available (whether one wants it or not) and it becomes inefficient to push through traditional deterministic computing paradigms.

### Conclusion

The results of this article show that stochastic computation provides an attractive framework for the investigation of computational properties of cortical microcircuits, and of networks of microcircuits that form larger neural systems. In particular it provides a new perspective for relating the structure and dynamics of neural circuits to their computational properties. In addition, it suggests a new way of understanding the organization of brain computations, and how they are modified through learning.

## Methods

### Network states and distributions of network states

#### Markov states

The Markov state 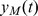 (or more explicitly, 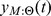) of a network at time 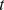 is defined here as the recent history of spike times of all neurons in the network within the period 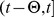. The term “Markov” refers to the fact that, under mild conditions and for a sufficiently long window 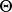, the network dynamics of a neural circuit after time 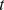 becomes independent of the network activity at times 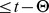, given the Markov state 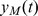 and the external input 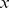. Hence, the network dynamics has the Markov property with respect to this state definition.

For each neuron 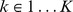 in a neural circuit a spike history of length 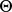 is defined as the list of spike times emitted by neuron 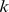 within the window 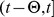. Spike times are counted relative to the beginning of the window at 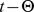. If 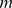 is the number of spikes within 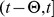 for neuron 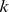, then the list takes the form,
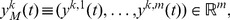
(3)where 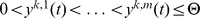.

We denote the space of all possible network states of length 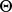 by 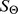 or, when unambiguous, simply by 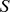. Note that this definition is equivalent to the state definition in [18], to which the interested reader is referred for further formal details (e.g. the associated 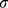-algebra 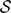 of the state space 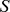).

#### Scope of theoretical results: Required properties of the network and neuronal noise models

We study general theoretical properties of stochastic spiking circuit models, driven by some external, possibly vector-valued, input 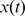, which could represent for example input rates in a set of input neurons or injected input currents. Formally, the input sequence can assume values from any state space 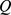; a concrete example is vector-valued input with 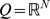, where 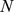 is the number of input dimensions.

We consider in this article two different noise models for a neuron: In noise model I, the spike generation is directly modeled as a stochastic process. All network dynamics, including axonal delays, synaptic transmission, short-term synaptic dynamics, dendritic interactions, integration of input at the soma, etc. can be modeled by a function which maps the Markov state (which includes the recent spike history of the neuron itself) onto an instantaneous spiking probability. This model is highly flexible and may account for various types of neuronal noise. In the more specific noise model II, the firing mechanism of the neuron is assumed to be deterministic, and noise enters its dynamics through stochastic vesicle release at afferent synaptic inputs. Also combinations of noise models I and II in the same neuron and circuit can be assumed for our theoretical results, for example neurons with a generic stochastic spiking mechanism which possess in addition stochastic synapses, or mixtures of neurons from model I and II in the same circuit.

In noise model I, the instantaneous spiking probability of neuron 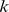 at time 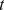 is given by,
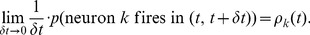
(4)This instantaneous firing rate 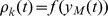 at time 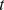 is assumed to be bounded and completely determined by the network's current Markov state 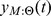, for some sufficiently large 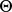. More precisely, the following four assumptions are made for noise model I:

*A*1 **Spikes are individual events:** We assume that,
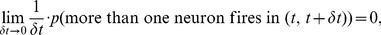
(5)which is, for example, fulfilled if each neuron has some independent source of stochasticity.

*A*2 **Bounded rates:** The instantaneous firing rates are bounded from above:


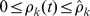
 for some 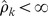. The ensuing upper bound on the total network firing rate is denoted by 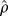, i.e. 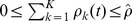. It is assumed that instantaneous rates are bounded at any time, and in the presence of any input 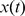.

*A*3 **Bounded memory:** The firing rates 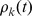 at time 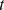 depend on the network's past activity only through the history of recent spikes in a finite window 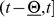 of length 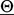. Hence, the *direct* effect of a spike at time 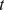 on future firing rates of all neurons is limited to a bounded “memory period”, 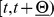. This bounded memory period 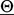 can be understood as a lower bound for 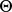 during the subsequent convergence proofs (since smaller 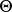 would violate the Markov property). In addition to this bounded-memory dependence on network spikes, 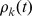 may depend on the current input 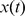 in any manner consistent with 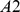.

*A*4 **Time-homogeneity:** The functional mapping from recent spikes and/or input signals 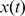 to instantaneous firing rates 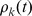 does not change over time. In particular, we do not consider long-term plasticity of synaptic weights and/or excitabilities in this work.

Assumptions 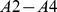 can be summarized as follows: Let 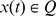 and 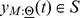 be the trajectories of input and network states as defined above. Then there exists a memory constant 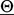 and rate bounds 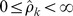, such that for each neuron 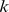 there exists a function 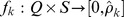, where 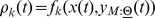 for all 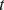. The function 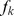 is time-invariant but otherwise unconstrained, and can capture complex dynamical effects such as non-linear dendritic interactions between synaptic inputs or short-term plasticity of synapses.

The input signal 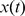 can formally represent any variable which exerts some arbitrary influence on the instantaneous network dynamics (the neuronal firing functions 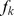). In the simplest case, 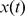 could be a vector of firing rates controlling the spiking behavior of a set of 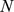 input neurons 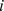, such that 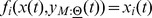 in these neurons. In this case (which we focused on in the main text), input neurons are formally considered part of the circuit 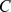. Note that in principle, 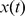 could also represent the strength of currents which are injected into a subset of neurons in the network 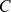, or the recent spiking history of a set of external input neurons (“input Markov states”). If the input comprises rates or currents, these can be either fixed (e.g. fixed input firing rates) or dynamically changing (in particular rates which are either subject to stochastic ergodic dynamics, or periodically changing rates). Below convergence proofs will be provided for both fixed and dynamic input conditions. If the input is defined in terms of input Markov states, the dynamic input analysis is applicable under conditions described further below.

In noise model II the basic stochastic event is a synaptic vesicle release (in noise model I it is a spike). Accordingly, the Markov state 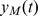 of a network in noise model II is defined as the list of vesicle release times for each synaptic release site in the network (instead of spike timings for each neuron). We assume here that each synaptic release site releases at a given instance 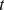 at most one vesicle filled with neurotransmitters. But a synaptic connection between two neurons may consist of multiple synaptic release sites (see [102], [103] and [3] for reviews). Instead of expressing the network dynamics through an instantaneous firing probability function for each neuron 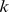, 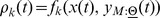 (noise model I), for noise model II the network dynamics is expressed in terms of instantaneous release probabilities for each synapse 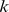: 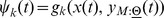. Similar to noise model I, it is assumed that there exists a window length 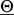, such that the dynamics of vesicle release at time 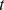 is fully determined by the timing of previous vesicle releases within 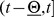, and hence can be expressed in terms of a corresponding variation of the definition of a Markov state 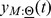. The same framework of assumptions applies as in noise model I: vesicle releases are individual events, and the functions 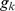 are assumed to be bounded from above by rate constants 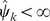.

Combinations of noise model I and II are also possible. In this case, the Markov state 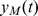 may contain both spike times and vesicle release times. The assumptions of noise model I/II described above apply to the corresponding stochastic neurons and vesicle releases, respectively. Altogether, note that all three types of networks (based on model I, II and mixtures of the two) are based on a common framework of definitions and assumptions: in all cases the dynamics is described in terms of stochastic components (neurons, synapses) which generate point events (spikes/vesicle releases) according to instantaneous probabilities which depend on the recent event history of the network.

#### Convergence of state distributions

Below, proofs for the existence and uniqueness of stationary distributions of network states for the considered network models are given. Furthermore, bounds on the convergence speed to this stationary distribution are provided. To obtain a comprehensive picture, convergence is studied under three different input conditions: constant, stochastic, and periodic input. All proofs are described in detail for noise model I. The results transfer in a straightforward manner to noise model II and mixtures of these two models, since the same framework of assumptions applies to all cases.

#### Network dynamics as a Markov process

We view the simulation of a cortical microcircuit model, under a given input condition and starting from a given initial network state, as a random experiment. Formally, we denote the set of all possible outcomes in this random experiment by 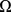, the set of all considered *events* by 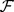 (i.e. a 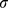-algebra on 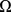), and the probability measure which assigns a probability to each event in 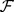 by 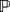. An outcome is the result of a single run of the network. An outcome is associated with an assignment of particular values to all defined random variables. An event is a set of outcomes, for example the set of all outcomes in which neuron 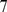 spikes within the first 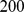 milliseconds of the experiment. Suppose 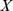 is a random variable with some state space 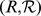, i.e. 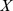 assumes values in 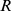, and 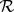 is a set of events on the space 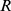. Formally, such a random variable 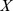 is defined as a map 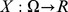, which assigns a value 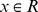 to every possible outcome 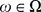. To denote the probability that the random variable 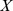 assumes some value in the set 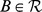, we define the short-hand 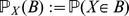. Furthermore, if 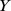 is another random variable we use the notation 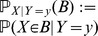 for conditional probabilities, and write even shorter, when unambiguous, 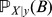. The base probability space 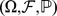 is assumed to be rich enough such that all random variables which are needed in the following exist.

We define the index set of time 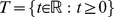, and the stochastic process 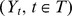, as a description of the stochastic evolution of Markov states of a network 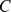 for 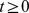. For each time 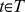 we define a random variable 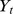 (also written 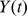) representing the Markov state of the network at time 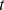. 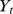 takes values on the state space 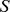 of all possible Markov states of some fixed duration 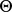. We denote by 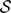 the 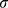-algebra associated with 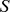. The assumptions on the network described in the previous section imply that the process has the Markov property for Markov states of any length 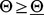, since the future evolution of the process is then entirely independent of the past, given the current Markov state. For the subsequent proofs, we therefore assume some 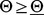. We also define a random variable 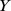 of *entire sample paths* on the measurable space 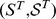, i.e. a map 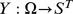. Realizations of 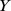 are sample paths (or trajectories), i.e. functions 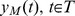, taking values in 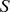. Since realizations of 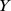 are functions, 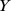 can be thought of as a random function.

For subsequent proofs the following definition of a *transition probability kernel* is essential: A transition probability kernel 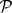 on a measurable state space 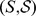 is a function 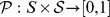, which assigns a probability to the transition from any point 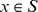 to any set 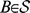. More precisely, if one fixes a particular “initial state” 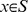, then 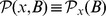 is a probability measure in its target argument 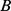, corresponding to the result of applying the transition kernel 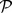 to 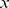 (in addition, for each event 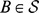 in the target space, 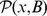 is 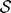-measurable in its source argument). Stochastic transition matrices of Markov chains are, e.g., transition probability kernels.

Here we write 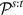 for the transition probability kernel corresponding to progression of the state of the network 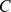 from time 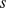 to 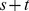, i.e.,
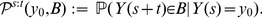
(6)We further define the shorthand 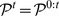 for the progression of duration 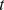 starting from initial time 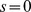. Transition kernels can also be applied to probability measures 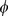 of initial states 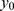 (as opposed to single initial states 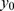). We will write 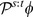 to denote the result of applying the kernel 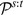 to an initial probability measure 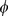. The result 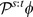 is again a probability measure, assigning a probability to any event 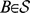 on the state space according to:
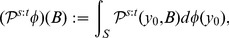
(7)Since 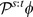 is again a probability measure on the state space 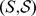, transition kernels can be applied sequentially. Note that due to the Markov property one has, 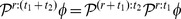 for 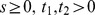.

#### Stochastic network dynamics is contracting

Before studying specific input conditions, a few basic key properties of the network dynamics 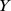 are developed. Let 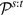 be the transition probability kernel corresponding to progression of the network 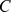 from time 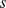 to 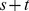. For the proofs below, transitions to the *resting state*, 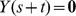, will be of particular importance. The resting state 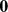 is defined as the “empty” Markov state in which no spikes occurred within the last 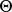 time units. The first key observation is the following Proposition:

**Proposition 1** *Consider the probability* 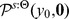 *, that the process* 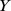 *will be in the resting state* 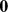 *at time* 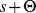 *, starting from some initial state* 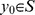 *at time* 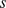 *. This “return probability” to the resting state is bounded from below by,*

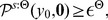
(8)
*where* 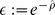 *. This holds regardless of the input trajectory* 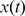 *driving the network.*

The proposition follows directly from the fact that 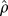 bounds the sum of all instantaneous firing rates in the network. Hence with at least probability 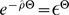 no neuron fires within 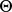 time units (cf. [18]). In technical terms, this implies that the stochastic kernel corresponding to a duration of length 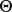 fulfills the Doeblin condition [104] – a property which is highly useful for proving convergence and ergodicity results.

Proposition 1 entails a central contraction property of stochastic networks of spiking neurons 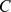, which holds in the presence of any input trajectory 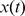, and forms the basis for several subsequent proofs. The following definitions are essential: We will measure below the difference between any two probability distributions 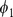 and 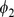 in terms of the total variation 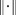 of the signed measure 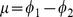. Any such signed measure 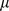 can be expressed in terms of its non-negative and non-positive components, 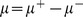, where 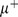 and 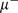 are both non-negative measures (but in general no probability measures). The total variation of a signed measure 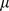 on a measurable space 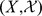 is defined as 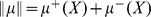, i.e. the total mass of its positive and negative components. According to this definition, 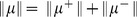.

**Lemma 1 (Contraction Lemma)** *The following strict contraction property holds for the Markov process* 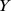 *, for any* 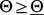 *, and for any initial probability measures* 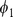 *and* 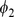 *at any time* 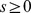 *:*

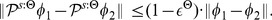
(9)
*In words: applying the dynamics of the network* 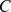 *for* 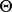 *time units is guaranteed to reduce the distance between any two initial distributions* 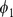 *and* 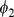 *of network states by a factor* 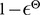 *.*

**Proof:** Define the auxiliary measure 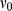 as zero everywhere outside 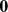, and 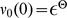. Rewrite 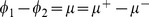 in terms of the non-negative measures 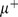 and 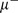, such that
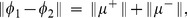
(10)and note that 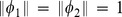 implies that 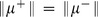. Then
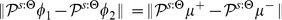
(11)

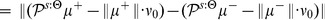
(12)

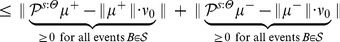
(13)

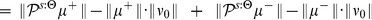
(14)

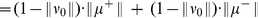
(15)

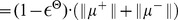
(16)

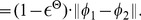
(17)The equality in (11) follows from linearity of transition probability kernels. The transition to (13) is an application of the triangle inequality. The transition to (14) uses the fact that both 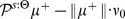 and 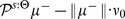 are non-negative: this follows from Proposition 1, which ensures that the measure 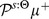 has at least mass 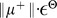 at the resting state 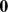 and, hence, for any (non-negative) measure 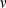,
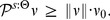
(18)Finally, note that (15) uses a general property of transition probability kernels 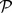, which ensures that 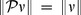, for any non-negative measure 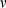.

Note that the above Contraction Lemma which holds for spiking neural networks has some similarities to Lemma 1 in [105] who analyzed artificial analog neural networks in discrete time.

#### Proof of Theorem 1 for fixed input rates

We divided the precise formulation of Theorem 1 into two Lemmata: Lemma 2 is a precise formulation for the case where inputs are fixed (e.g. fixed input rates). Lemma 3 in the next section corresponds to the case where input rates are controlled by a Markov process. The precise assumptions on the network model required for both Lemmata are described above (see “Scope of theoretical results”).

Here we assume that the vector of inputs 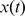 provided to the network is kept fixed during a trial. Concretely, this is for example the case if there is a set of input neurons whose rates are fixed. In this case, 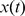 is a vector of input rates, which remains constant over time. The input neurons are formally considered part of the network in this case. Alternatively, a constant 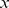 could correspond to constant currents which are injected into a subset of neurons.

Under constant input conditions, 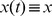, the dynamics of the process is time-homogeneous: the transition probability kernels are invariant to time-shifts, i.e.
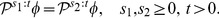
(19)


**Lemma 2** *Let* 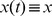 *. Then the Markov process* 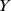 *has a unique stationary distribution* 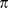 *, to which it converges exponentially fast,*

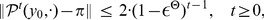
(20)
*from any initial Markov state* 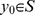 *.*

**Proof:** 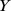 is clearly non-explosive, aperiodic and stochastically continuous (cf. [18]). To prove exponential ergodicity it thus suffices to show that some skeleton chain is geometrically ergodic (see for example Theorem 18.1 in [106]). The skeleton chain 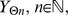 with transition probability kernel 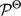 is aperiodic and irreducible and hence has a unique stationary distribution 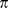. Then, through recursive application of Lemma 1 with 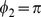,
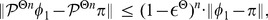
(21)

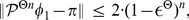
(22)proving geometric ergodicity of the skeleton chain, and thus exponential ergodicity of 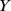. The quantitative convergence bound follows from (22) by choosing a singleton 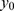 as initial distribution, and using the general fact that for any transition probability kernel 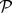 and distributions 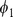 and 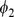,
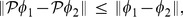
(23)thus guaranteeing that the total variation distance does not (temporarily) grow between 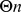 and 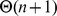. 

Lemma 2 provides a general ergodicity result for the considered class of stochastic spiking networks in the presence of fixed input rates 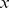. The proof relies on two key properties of stochastic spiking networks: aperiodicity and irreducibility. These properties can be understood intuitively in the context of Figure 1H. If the intrinsic network dynamics was not aperiodic, for example, then one might be able to observe oscillating pattern frequencies over time (as in Figure 4C). Lemma 2 proves that this cannot occur in stochastic spiking networks as long as input rates are fixed. Oscillating pattern frequencies can indeed only emerge when input rates are themselves periodically changing (see Theorem 2 and Figure 4). If the network dynamics was not irreducible on the other hand, i.e. if there were network states which are unreachable from some other network states, then pattern frequencies could potentially be observed to converge to different fixed points for different initial states (e.g. the two lines in Figure 1H settling at different values). This cannot occur in stochastic spiking networks due to Proposition 1 which guarantees that the state space is connected through the resting state 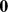.

Note that, although aperiodicity and irreducibility are well known necessary and sufficient conditions for ergodicity in discrete time Markov chains on finite state spaces, they are not sufficient for exponential ergodicity in continuous time Markov processes on general state spaces (see [107] for precise definitions of 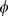-irreducibility and aperiodicity for such processes). Additional conditions in this more complex case which ensure exponential ergodicity, such as nonexplosivity, stochastic continuity and geometric ergodicity of a skeleton chain, have also been taken into account in the proof for Lemma 2 (i.e. stochastic spiking networks also meet these additional criteria).

Lemma 2 constitutes a proof for Theorem 1 for fixed input rates 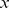. In the main text we refer to the stationary distribution of the circuit 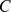 under fixed input 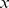 as 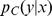. The proof above guarantees a stationary distribution for both Markov and simple states. In the main text 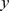 refers to the simple network state 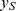 if not stated otherwise.

#### Proof of Theorem 1 for input rates controlled by a Markov process

Fixed input assumptions may often hold for the external input 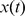, driving a stochastic computation in a neural system 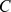, only approximately. Stochastic fluctuations on various spatial and temporal scales may be present in the input. In addition, inputs may have their own short-term stochastic dynamics: Imagine, for example, a visual scene of randomly moving dots. Despite the presence of such short-term dynamical features in the input, in many cases one may still suspect that network state distributions converge. Indeed, below we generalize the convergence results from the constant case to the quite large class of stochastic (and stochastically changing) inputs which are generated by a uniformly ergodic Markov process. Uniform ergodicity is defined as exponential ergodicity (exponentially fast convergence to a unique stationary distribution) with convergence constants which apply uniformly to all initial states [107] (this holds for example for the convergence constants in Lemma 2).

Let 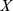 be a time-homogeneous *input Markov process*, in the sense that the input trajectory 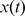 provided to the network 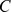 is itself generated randomly from a Markov process 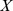. Let 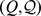 be the (measurable) state space of 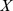. Then define a joint input/network Markov process 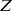 on the state space 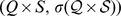, where 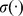 denotes the 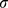-algebra generated by . Further definitions for 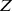 are analogous to those introduced for 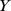.

**Lemma 3** *If the input process* 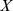 *is uniformly ergodic, then the joint Markov process* 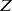 *has a unique stationary distribution* 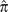 *on the joint input/network state space, to which convergence occurs exponentially fast, i.e. there exist constants* 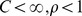 *, such that*

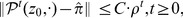
(24)
*for any initial state* 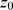 *of the joint Markov process* 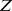 *.*

**Proof:** If 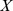 and 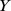 were entirely independent processes (if 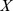 did not influence 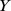) then the joint process 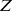 would automatically be exponentially ergodic if both 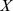 and 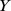 are. Although in the present case 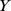 is not independent of 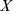, a weaker version of independence applies: the return probability to the resting state 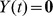 during 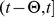 is at least 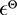 *regardless* of the input trajectory of 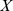 during that time. This property can be exploited to show that the distribution of hitting times to a joint resting state has an exponential bound. It follows that the joint process is exponentially ergodic. A detailed proof is given in the next section. 

The second part of Theorem 1 (exponentially fast convergence for the case of external input generated by an ergodic Markov process) follows from Lemma 3. Note that in the main text we slightly abuse the notation 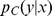 for the dynamic case to indicate the stationary distribution over network states 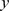, where 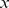 denotes a specific Markov process controlling the inputs.

#### Detailed proof of Lemma 3

We have split the proof of Lemma 3 into proofs of four auxiliary claims (Propositions 2–5). Consider the following variations of Proposition 1, which hold for the Markov process 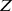 describing the joint dynamics of input and network states. Let 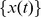 denote a particular input sequence defined for 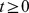 (a realization of the input process 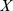) and 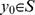 an initial network Markov state (with 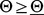) at time 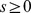. Then
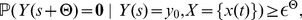
(25)

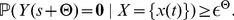
(26)


It is easy to show that these properties, together with the fact that 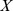 is uniformly ergodic, ensure that 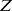 is irreducible and aperiodic. Hence, to prove exponential ergodicity of 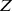 it suffices to show that some skeleton chain is geometrically ergodic [107]. To that end, we will consider the skeleton chain 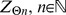 and prove geometric ergodicity by showing that the hitting time distribution 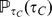 to a *small set* 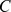 on the joint state space 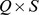 of input and network states admits an exponential bound.

The *hitting time* 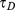 to some set 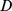 on the input state space 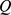 is defined as
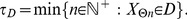
(27)For notational convenience we abbreviate in the following 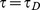. Due to uniform ergodicity of 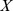 (which implies Harris recurrence [107]), there exists some set 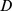 to which the hitting time 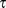 is finite (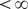) from any initial state, with probability one [108]. Furthermore, there exists according to [107] a *small set* 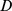 and constants 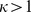 and 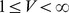, such that
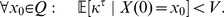
(28)This implies that there exists a small set 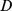 on the input state space 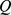 which can not only be reached in finite time from any initial input state 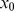, but for which the hitting time distribution to 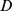 has also finite mean and variance (and finite higher-order moments). At least one pair of constants 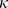 and 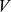 which fulfills (28) is guaranteed to exist, but in fact the following Proposition shows that one can specify a particular desired bound on the right-hand side (for reasons which will become clear later), and find a matching 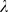 on the left-hand side.

**Proposition 2** *There exists a* 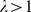 *, such that*

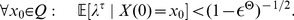
(29)


**Proof:** Define 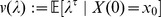. Let 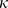 and 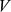 be any valid pair of constants which fulfills (28). The trivial case is 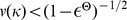. In the remainder of the proof it is assumed that 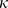 is “too large”, such that 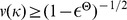. By definition of the exponential function, for any 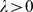,
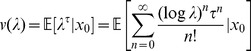
(30)

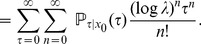
(31)By Tonelli's theorem, since all summands are non-negative, the order of the double sum can be exchanged:
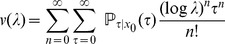
(32)

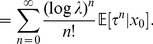
(33)Note that 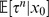 are the moments of the distribution 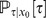. By uniform ergodicity of 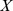, all moments must exist, and in addition there exists a 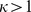 such that 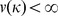. It is straightforward to see that the series then converges for all 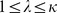, such that 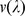 is continuous on 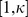. Finally, since 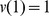 and 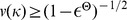, by the intermediate value theorem there exists some 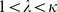 such that 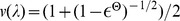.

Denote by 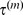 the time at which the skeleton chain 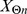 visits the small set 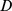 for the 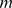-th time:
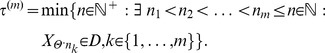
(34)Furthermore, denote by 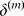 the time between the 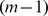-th and 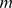-th visit:
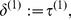
(35)

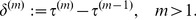
(36)


According to this definition, one can express the hitting time of degree 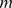 as 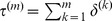. The following Proposition extends the exponential bound on the first hitting time to hitting times of higher degrees.

**Proposition 3** *There exists a* 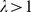 *, such that,*

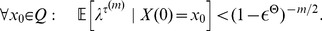
(37)


**Proof:**

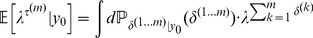
(38)

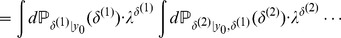


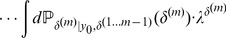
(39)

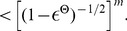
(40)


Let 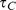 be the hitting time to the small set 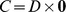 on the joint state space 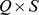 of input and network states,
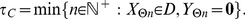
(41)


Furthermore, let 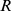 be the number of visits to the small set 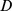 prior to and including time 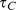,
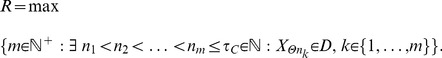
(42)


**Proposition 4** *For any input trajectory* 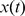 *and any initial network state* 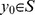 *,*

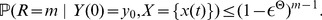
(43)


This follows from (25) and (26) which ensure that whenever the input process visits the small set 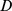, there is also a small probability that the network is in the resting state.

**Proposition 5** *There exists a* 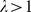 *and a constant* 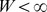 *such that,*

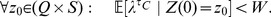
(44)


**Proof:** Let 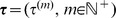. Choose some 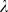 which fulfills Proposition 3.
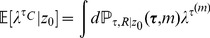
(45)

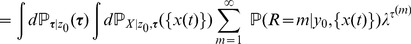
(46)

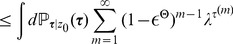
(47)

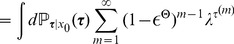
(48)

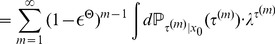
(49)

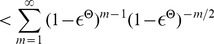
(50)

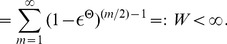
(51)


By Proposition 5, 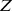 is exponentially ergodic [107]. This completes the proof of Lemma 3.

#### Distribution of trajectories of network states

The Markov states 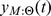 are segments of spiking trajectories of length 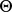. Hence, all statements developed above apply to convergence of the distribution over these (short) spiking trajectories. If one is interested in the convergence of longer trajectories, the simplest option is to choose a larger 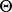, since any finite 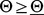 is admissible, and all convergence results readily extend to trajectories of any finite length. A limitation of this approach is that the quantitative convergence statements will suffer from making 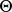 too large, since convergence rates scale approximately with 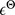 (and 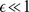). Hence, in practice, empirical convergence tests are required to make statements about specific circuits.

### Proof of Theorem 2

If the input sequence is periodic with period 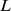, i.e. 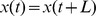 for all 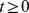, then the Markov process 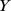 will be time-periodic, in the sense that transition kernels are invariant to shifts which are multiples of the period 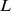:
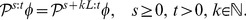
(52)


This implies the following result, which is a more precise version of Theorem 2:

**Lemma 4** *Under periodic input, i.e.* 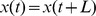 *for all* 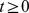 *with some* 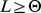 *, the time-periodic Markov process* 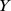 *with period* 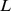 *has a periodically stationary distribution* 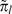 *, to which convergence occurs exponentially fast from any initial state. In particular, for every* 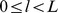 *there exists a unique stationary distribution* 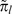 *such that,*

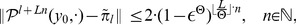
(53)
*from any initial Markov state* 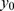 *.*

**Proof:** For each 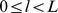 there exists a skeleton chain 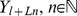, with transition probability kernel 
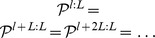
, which is time-homogeneous, irreducible, and aperiodic and thus has a unique stationary distribution 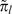. An application of 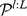, which corresponds to a full period, decreases the total variation distance to 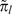 by at least 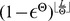:
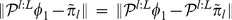
(54)

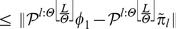
(55)

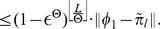
(56)The first inequality follows from the fact that applying the remaining 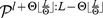 can only further decrease the total variation distance between the two distributions, according to (23). The second inequality is due to Lemma 1.

Lemma 4 then follows from recursive application of (54)–(56) for multiple periods, and choosing a singleton 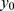 as initial distribution.

In the main text, we use the notation 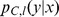 for a phase-specific stationary distribution, where 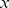 denotes a specific periodic input sequence.

### Relation to previous theoretical work

Previous work on the question whether states of spiking neural networks might converge to a unique stationary distribution had focused on the case where neuronal integration of incoming spikes occurs in a linear fashion, i.e., linear subthreshold dynamics followed by a single output non-linearity [17], [18]. In addition these earlier publications did not allow for the experimentally observed short term dynamics of synapses. The earlier publication [17] had studied this question as a special case of the mathematical framework of non-linear Hawkes processes, a class of mutually exciting point processes (see also [109]). The authors had arrived for the more restricted type of neurons which they considered at exponential convergence guarantees under a similar set of assumptions as in this article, in particular bounded memory and bounded instantaneous firing rates (and these results can thus be seen as a special case of Theorem 1, for the case of constant external input). [17] also derived convergence results for linearly integrating neurons with unbounded memory dynamics under a different set of assumptions, in particular Lipschitz conditions on the output non-linearity and constraints on the effective connectivity matrix of the network. Whether such alternative set of assumptions can be found also in the context of non-linear integration of incoming spikes (needed e.g. for synaptic short-time dynamics or dendritic non-linearities) remains an open question.

The recent publication [18] also focused on neurons with linear sub-threshold dynamics followed by an output non-linearity (termed there non-linear Poisson neurons) with static synapses, and extended the convergence results of [17] to networks with Hebbian learning mechanisms. In addition, an important methodological innovation by [18] was the introduction of spike history states (which are equivalent to the Markov states 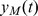 in this article) which allowed them to study convergence in the framework of general Markov processes (in contrast to point processes in [17]). Theorem 1 in this article contains as a special case the convergence results of [18] for their Model I (non-linear Poisson neurons in the absence of Hebbian learning). We note that although [18] focused on neurons with linear sub-threshold dynamics (and required that firing rates are strictly greater than 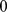), their method of proof for Model I could be readily extended to cover also non-linear sub-threshold dynamics to yield the first part of our Theorem 1 (the case where inputs have constant firing rates).

We are not aware of previous work that studied convergence in spiking networks with dynamic synapses, or in the presence of stochastic or periodic inputs (see the second part of Theorem 1 concerning Markov processes as input, and Theorem 2). We further note that our method of proof builds on a new and rather intuitive intermediate result, Lemma 1 (Contraction Lemma), which may be useful in its own right for two reasons. On the one hand it provides more direct insight into the mechanisms responsible for convergence (the contraction between any two distributions). On the other hand, it holds regardless of the input trajectory 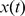, and hence has in fact an even larger scope of applicability than Theorem 1 and 2. Hence, Lemma 1 could be, for example, applied to study non-stationary evolutions of state distributions in response to arbitrary input trajectories.

### Extracting knowledge from internally stored distributions of network states

A key advantage of sample-based representations of probability distributions is that probabilities and expected values are in principle straightforward to estimate: To estimate the expected value 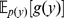 of a function 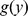 under a distribution 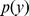 from a number of samples 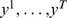, simply apply the function to each sample and compute the time average 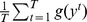. As long as the samples 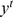 are distributed according to 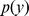, either independently drawn, or as the result of an ergodic Markov chain/process with stationary distribution 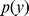, this estimate is guaranteed to converge to the correct value as one increases the number of samples [110], i.e. 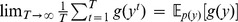. Estimates based on a finite observation window represent an approximation to this exact value.

Under the mild assumptions of Theorem 1 the dynamics of a stochastic spiking network in response to an input 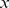 are exponentially ergodic and there exists a unique stationary distribution 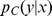, according to which network states 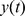 are distributed. Hence, the expected value 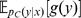 of any function 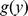 under the stationary distribution 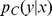 can be estimated by computing the sample-based time average
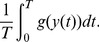
(57)


This approach can also be used to estimate marginal probabilities, since probabilities can be expressed as expected values, for example,
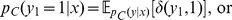
(58)

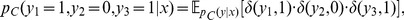
(59)where 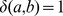 if 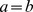 and 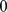 otherwise. Hence, in order to estimate the probability 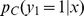 it suffices to measure the relative time the neuron spends in its active state, i.e. 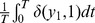. Similarly, to estimate the probability 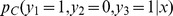 it is sufficient to keep track of the relative frequency of the pattern 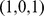, by computing 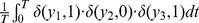.

### Simulations of data-based cortical microcircuit models

All simulations of microcircuit models for Figures 1–4 were carried out in PCSIM [111]. A time step of 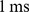 was chosen throughout. Further analysis of spike trains was performed in Python [112].

#### Stochastic neuron model

A stochastic variation of the leaky integrate-and-fire model with conductance-based integration of synaptic inputs was used, for both excitatory and inhibitory neurons. Sub-threshold dynamics of the membrane potential 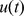 was defined according to a standard leaky integration model with conductance-based synapses [113], using passive membrane parameters 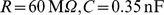 and a resting potential 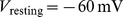. At simulation start, initial potentials were randomly chosen from 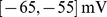. Reversal potentials for excitatory synapses and inhibitory synapses were set to 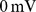 and 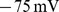, respectively. Neuronal noise was modeled by a voltage-dependent instantaneous probability of firing (instead of a fixed threshold) [48],
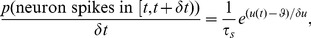
(60)for 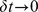, with parameters 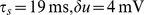 taken from [48]. In contrast to [48] we used a non-adaptive threshold, 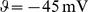. After a spike, a neuron enters an absolute refractory period of 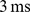. Thereafter, the membrane is reset to the resting potential and leaky integration is continued. Altogether, the resulting neuronal spiking mechanism is consistent with the theoretical noise model I described in equation (4).

Note that Theorem 1 also holds for substantially more complex multi-compartment neuron models incorporating, for example, data on signal integration in the dendritic tuft of pyramidal cells [114], [115], and data on 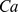-spikes in pyramidal cells on layer 5 [116], but we have not yet integrated these into the simulated microcircuit model because of a lack of coherent quantitative data for all the neuron types involved.

#### Synaptic short-term plasticity

The short-term dynamics of synapses in all data-based simulations was modeled according to [47], [117]. The model predicts that at a synapse with “weight” 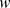 the amplitude 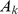 of the 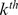 spike in a spike train with interspike intervals 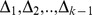 is given by,
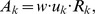


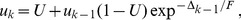
(61)

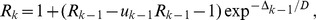
where the hidden dynamic variables 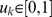 and 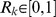 are initialized for the first spike to 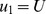 and 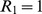. The parameters 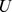, 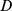 and 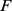 represent the utilization of the synaptic efficacy of the first spike after a resting state, the recovery and the facilitation time constants, respectively. These parameters were set based on experimental data on short-term plasticity in dependence of pre- and post-synaptic neuron (excitatory or inhibitory) as in [30] (see in particular Table 1 in this reference), by randomly drawing for each neuron values for 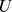, 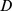, and 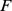 from corresponding data-based Gaussian distributions.

**Table 1:** Number of randomly chosen neurons per pool for readout neuron in Figure 2G.

	E	I
L2/3	120	30
L4	80	20
L5	200	50

#### Connectivity and synaptic parameters

Synaptic parameters and connectivity rules for the data-based cortical column model were taken from [30], see Figure 1A. In particular, we adopted from [30] the connection probabilities and transmission delays for each type of connection (EE, EI, IE, II) and each cortical layer ([30], Figure 1), as well as short-term plasticity parameters. Furthermore, synaptic efficacies of individual synapses were drawn from Gamma distributions with data-based means and variances for each type of connection (EE, EI, IE, II) taken from [30]. Two input streams were connected to the microcircuit, each consisting of 40 input neurons. In contrast to [30] we used rate-based Poisson input neurons instead of injecting “frozen” spike patterns. Background synaptic inputs were emulated as in [30] via background input currents to each neuron, with conductances modeled according to [118]. To adjust connectivity for cortical microcircuit models of different sizes, we also adopted the method proposed by [30], in which recurrent weights are scaled inversely proportional to network size.

We tested the validity of our cortical microcircuit model by comparing the average activity of different layers (see Figure 2A) under various conditions against the values reported by [30]. We confirmed that all layers exhibited very similar average activity to [30] under all considered conditions.

### Details to small microcircuit model in Figure 1

The small cortical microcircuit model of Figure 1B was constructed based on the cortical column template of [30]: Synaptic connections between neurons and their weights were chosen to approximately reflect connection probabilities and mean synaptic strengths of the cortical column template [30]. Due to the very small size of this network, the resulting dynamics was not immediately satisfactory (for example, the influence of inputs on Layer 5 neurons was too weak). To shift the circuit into a more responsive regime, we manually adjusted a few synaptic weights and neuronal excitabilities. In particular, we injected small constant currents into some of the neurons to modulate their intrinsic excitability. Furthermore, to increase activity and correlations between highlighted neurons 2, 7 and 8, we increased synaptic weights 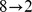 and 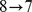 by factors 5 and 10, respectively. To set the initial Markov state of the network, preparatory input was shown for 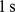 before the actual start of the simulation. Two different preparatory inputs were injected to set the two initial states considered in Figure 1F–H (first: 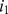 at 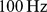, 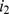 at 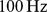, second: both 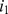 and 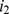 at 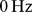). To reproduce the same initial Markov state in multiple trials (for example the two trials shown in Figure 1F), the same random seed was used during the preparatory phase for these trials. The random seed was then reinitialized at 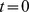 to different values for each trial.

### Estimates of required computation time

#### Gelman-Rubin univariate and multivariate analysis

Various methods have been developed for measuring convergence speed to a stationary distribution in the context of Markov chain Monte Carlo sampling [56], [119], [120]. The Gelman Rubin diagnostic, which we adopted in this article, is one of the most widely used methods [55], [57], [58], [119], besides other popular methods such as the diagnostics by Raftery and Lewis [121] and by Geweke [122]. We remark that the consensus in the literature is that no single method is perfect in general. Some attractive properties of the Gelman Rubin method are general applicability to any MCMC system (some other methods only work, for example, in the context of Gibbs sampling), ease of use, ease of implementation, computational efficiency, and the fact that results are quantitative (in contrast to graphical diagnostics) [56], [119].

The Gelman-Rubin convergence diagnostic [55] takes as input samples from 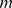 different runs (trials/chains/sequences) produced by the same system, started from different initial states. The method was originally developed for discrete-time systems in the context of Markov Chain Monte Carlo sampling. Our simulations use a time step of 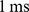, so we simply treat each simulation step as one discrete time step in a Markov chain. The Gelman-Rubin method produces as output the potential scale reduction factor 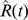 as a function of time 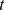. The scale reduction factor 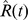 is an indicator for whether or not the system has converged at time 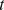. High values 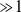 indicate that more time is needed until convergence, while values close to 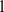 suggest that convergence has (almost) taken place.

For computing the scale reduction factor 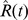 at time 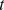, samples from the period 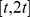 from each run of the network are taken into account. In the univariate case one focuses on a particular single variable (such as the marginal simple state of a single neuron, or the simple state of a “random readout” neuron as in the solid lines of Figure 2G). Let 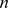 be the number of samples obtained from the period 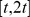 from each of the simulations. Then one defines
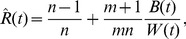
(62)where 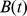 and 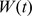 are between and within-sequence variances, respectively, which can be computed as described in [55], based on samples taken from the time period 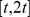. In the rare event of 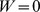, which happens for example if a neuron never fires and hence its state is constant across all runs, we set 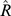 to 1.

An unfortunate source of confusion is the fact that Gelman and Rubin [55] originally introduced 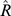 in its “variance” form equivalent to equation (62), but later in [57], [60] altered this definition and defined 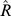 as the square root of (62). This issue is particularly critical when considering threshold values for 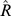: a threshold of 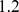 was suggested in the context of the original definition [59]. Later, a typical threshold of 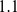 was suggested, but this lower threshold applied to the modified definition [57], [60]. Squaring this apparently lower threshold yields again a typical threshold of approximately 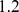.

In the multivariate case (e.g. when analyzing convergence of the vector-valued simple state of a small subset of neurons as in the dotted lines of Figure 2G) one takes vector-valued (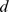-dimensional) samples, and computes the multivariate potential scale reduction factor 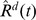 according to:
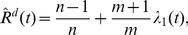
(63)where 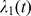 is the largest eigenvalue of 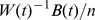, and 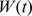 and 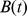 denote within and between sequence covariance matrix estimates (see [123] for details).

#### Convergence analysis for cortical microcircuit models

Gelman-Rubin values were calculated based on 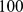 runs, where the duration of each run was 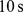 of biological time. We tried also much longer simulations of 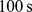 but did not notice any sign of non-convergent behavior. A random initial state was set in each run by showing random input for 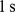 before the start of the actual simulation. This initial random input was fed into the network via the two regular input streams (40 neurons each), by assigning to each input neuron a random rate drawn uniformly from a 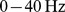 range. Convergence analysis of marginals was performed by applying univariate analysis to single components of the simple state 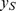, with 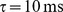. From individual marginal convergence values, mean and worst marginal convergence (as in Figure 2E,F) were derived by taking at time 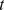 the mean/max over all individual 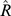-values at time 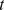. For pairwise spike coincidences (see Figure 2D), we analyzed samples of the product of simple states of two neurons (the product equals 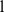 only if both neurons spiked within the last 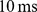).

Random readouts for Figure 2G were implemented by adding an additional excitatory observer neuron to the network which receives synaptic inputs from a random subset of 500 network neurons (we kept this number 500 fixed across simulations with different network sizes to allow a fair comparison). The number of randomly chosen neurons from each of the pools is given in Table 1.

Synapses onto the readout neuron were created in a similar manner as connections within the cortical column model: short-term plasticity parameters were set depending on the type of connection (EE or IE) according to [30]. The weights for EE and IE connections were randomly chosen from a Gamma distribution with mean 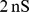 and scale parameter 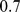, and mean 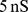 and scale parameter 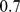, respectively. Gelman-Rubin convergence of readouts was then computed as for the marginal case.

Convergence analysis of vector-valued simple states of subsets of neurons (see Figure 2G) was performed by applying multivariate analysis to randomly chosen subnetworks of the cortical column. In particular, we randomly drew 5 neurons from each of the 6 pools, yielding a subnetwork of 30 neurons, and calculated 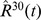.

### Impact of different dynamic regimes on the convergence time

In Figure 3 we compared convergence times in four different neural circuits. The first circuit was identical to the *small cortical microcircuit* from Figure 1. For the remaining three circuits, the same stochastic point neurons and conductance-based dynamic synapses with delays were used as for the data-based cortical microcircuit model. Dynamic synaptic parameters were set to the corresponding mean values of parameters used in the cortical column model. Synaptic delays of 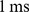 were used for all networks, except for the network with sequential structure (Figure 3C) where delays were 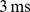. To modulate the intrinsic excitability of neurons we injected small currents to each neuron. The strengths of injected currents and connections were tuned for each network until the desired network activity was achieved. Synaptic background inputs were injected as in the cortical microcircuit model. To set different initial states (needed for Gelman Rubin analysis), during a preparatory phase of 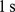 we injected into each neuron a random current chosen from 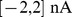. These small random input currents were strong enough to yield sufficiently diverse initial states. Gelman-Rubin values were then calculated based on 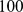 runs, where the duration of each run (after the preparatory phase) was 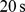 of biological time. Convergence analysis was performed on marginals (individual simple states with 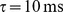). Mean and worst marginals were computed as described in the previous section. 

Below are additional details to the circuits used for Figure 3B–D: *The sparsely active network* of Figure 3B comprises one excitatory (E) and one inhibitory (I) population (each 10 neurons). Connections between neurons were drawn randomly according to the following set of connection probabilities: EE = 0.1, EI = 0.1, II = 0.9, IE = 0.9. *The network with sequential structure* of Figure 3C consists of two interconnected subnetworks where each one of them produces a stereotypical trajectory. Each subnetwork consists of a trigger neuron, a subsequent chain of neurons, and a pool of inhibitory neurons. Shown in Figure 3C are only the excitatory chain neurons from each subnetwork (neurons 1–15: first subnetwork; neurons 16–30: second subnetwork). Each excitatory neuron in the chain projects to all other neurons in the same chain with synaptic strengths decreasing with distance according to 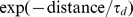 where 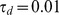 applies to the forward direction in the chain and 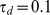 to the backward direction. The trigger neuron projects (forward) to the chain in the same fashion with 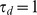. All neurons in the chain project to the inhibitory pool, and all neurons in the inhibitory pool project back to the trigger neuron and to the chain. Finally, the two subnetworks are combined such that the inhibitory pool of one subnetwork projects to the trigger neuron and the chain of the other subnetwork, and vice versa. This ensures that only one of the two subnetworks can be active at a time (competition between two trajectories). *The bistable network* of Figure 3D consists of two populations which strongly inhibit each other (each population comprising 10 neurons).

### Distributions of network states in the presence of periodic network input

The theoretical proof for Theorem 2 can be found after the proof of Theorem 1 above. For Figure 4F, a single long simulation (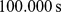) of the bi-stable network in Figure 4E was carried out. Each of the two pools was defined active at time 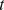 if more than two neurons from the pool had an active simple state at time 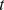 (with 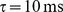). A transition was defined as the succession of a period in which one pool was active and the other pool inactive by a period in which the other became active and the first pool turned inactive. Between those two periods it typically occurs that either both pools are active or both are inactive for some short time. The exact time (and phase within the current cycle) of each transition was defined as the point in the middle of this intermediate period.

### Solving constraint satisfaction problems in networks of spiking neurons

#### Formulation of Sudoku as a constraint satisfaction problem

A constraint satisfaction problem consists of a set of variables defined on some domain and a set of constraints, which limit the space of admissible variable assignments. A solution to a problem consists of an assignment to each variable such that all constraints are met. To formulate Sudoku as a constraint satisfaction problem, we define for each of the 81 fields (from a standard 9×9 grid), which has to be filled with a digit from 1 to 9, a set of 9 binary variables (taking values in 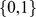) [124]. Each of these binary variables votes for exactly one digit in a field. The rules of the Sudoku game impose constraints on groups of these variables, which can be classified into the following three types.

*Given number constraints:* The given numbers of a puzzle are fixed. Hence, the binary variables for the given fields are constrained to fixed values, for example, a given value 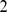 corresponds to fixed binary values 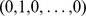.

*Unique field constraints:* In a correct solution, there must be only one digit active in each field. Hence in each field, exactly one of the 9 associated binary variables must be 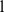, and all others must be 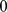 (equivalent to stating that the sum over these binary variables must equal 1).

*Unique group constraints:* There are three types of groups: rows, columns and 3×3 subgrids. There are 9 row groups, 9 column groups, and 9 subgrid groups. In any of these groups, each digit 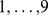 must appear only once. Hence, in each group, all binary variables voting for the same digit 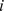 must sum to 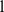.

#### Network architecture for solving Sudoku

Sudoku can be implemented in a spiking neural network by creating for each of the 9 binary variables in each Sudoku field a local group of 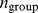 pyramidal cells. Whenever one of these pyramidal cells fires, the corresponding binary variable is set to 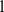 for a short period 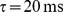. The binary variable is defined 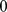 only if no neuron in its associated group fired within the last 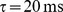. This mapping allows one to readout the current (tentative) solution represented by the network at any time 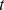. The tentative solution is correct only if all constraints are met. For all simulations we used 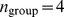, resulting in a total 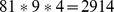 pyramidal cells. Constraints among Sudoku variables can be implemented via di-synaptic inhibition between the groups of pyramidal cells as detailed below.

*Given number constraints* are implemented by providing strong positive input currents selectively to those neurons which code for the given numbers, and negative currents to neurons coding for wrong digits in a given field. *Unique field constraints* are implemented by forming a winner-take-all (WTA) circuit among all 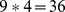 neurons associated with the same Sudoku field. A WTA circuit is modeled by a single inhibitory neuron which is reciprocally connected to all 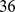 pyramidal cells. To reduce the probability that no pyramidal cell fires (which would violate the unique field constraint), thresholds of pyramidal cells are set to low values (see next section for details). *Unique group constraints* are implemented by a WTA circuit in which all neurons in a group which code for the same digit participate. In summary, there are 81 unique field constraints and 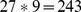 unique group constraints (in each group there is a constraint for each digit), yielding a total of 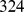 WTA circuits. These WTA circuits are partially overlapping, in the sense that each pyramidal cell participates in 4 of these WTA circuits (one for the unique value constraint in its field, and three for the unique group constraints in its row/column/subgrid).

Stochastic spike generation in both excitatory and inhibitory neurons is implemented consistent with the theoretical noise model I (see next section for details). The network thus fulfills all theoretical conditions for Theorem 1, and is guaranteed to have a unique stationary distribution 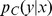 of network states, to which it converges exponentially fast. This landscape will have automatically peaks at those states of the network which fulfill most of the game constraints, since each of the WTA circuits ensures that invalid configurations with respect to that constraint are unlikely to occur. Any specific Sudoku problem can be set by providing input 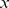 to the network in the form of strong currents to those neurons which correspond to the given values. This automatically modifies the landscape of the stationary distribution 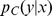 such that only (or predominantly) solutions consistent with the givens are generated. Finally, due to neuronal noise the network can quickly probe different peaks in the landscape (different promising solution candidates) and escape them equally fast. Importantly, this process may occur at different places in the Sudoku puzzle simultaneously. Hence, one can interpret the network dynamics also as a highly parallel stochastic search algorithm.

#### Details to implementation and simulations for Figure 5

Simulations for Figure 5 were performed in NEVESIM, an event-based simulator for networks of spiking neurons developed in C++ with a Python Interface [125]. The puzzle in Figure 5A was generated and rated “hard” by “Sudoku Solutions” [126]. Spike generation is modeled according to equation (60), with parameters 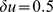, 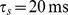. The stochastic threshold 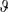 was set to 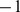 and 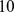 for excitatory and inhibitory neurons, respectively. An absolute refractory period of 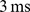 was chosen for pyramidal cells. To maximize the speed up of event-based simulations, PSPs were modeled in a simplified manner as current-based rectangular pulses of length 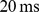 (in contrast to the more complex conductance based integration of synaptic inputs used for cortical microcircuit models).

WTA circuits were formed by reciprocally connecting a single inhibitory neuron to all participating pyramidal cells. The single inhibitory neuron was modeled to mimic the response of a population of inhibitory neurons (i.e. strong inhibition for a prolonged amount of time), using an absolute refractory period of 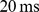, and strong bidirectional connections from and to excitatory neurons (synaptic weights 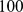 and 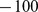, respectively).

To set a particular puzzle, given numbers were fixed by providing strong input currents to the corresponding pyramidal cells. In particular, neurons coding for the given numbers in a Sudoku field received a constant positive input current (a constant input 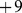 on the membrane potential). Neurons coding for conflicting digits in given Sudoku fields received a constant negative input current of strength 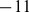.

A final practical remark concerns the number of neurons coding for each binary variable, 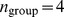. We found that networks with 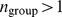 have a number of attractive properties compared to networks with single neuron coding. In particular firing rates of individual neurons can be lower (for 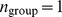 a pyramidal cell would need to constantly burst to indicate a steady active state). Also, synaptic efficacies among neurons can be made weaker, and overall spike response patterns appear more biologically plausible. In view of a potential implementation in analog neuromorphic hardware, population coded variable assignments are also less prone to single unit failures or device mismatch.
